# Serum proteomics analysis of lung transplant patients receiving different induction therapies

**DOI:** 10.3389/fimmu.2025.1616781

**Published:** 2025-08-28

**Authors:** Shahrooz Nasrollahi-Shirazi, Markus Unterwurzacher, Hatice Oya Berezhinskiy, Sophia Alemanno, Konrad Hoetzenecker, Clemens Aigner, Peter Jaksch, Thomas Mohr, Klaus Kratochwill, Alberto Benazzo

**Affiliations:** ^1^ Department of Thoracic Surgery, Medical University of Vienna, Vienna, Austria; ^2^ Comprehensive Center for Chest Diseases (CCCD), Medical University of Vienna, Vienna, Austria; ^3^ Division of Pediatric Nephrology and Gastroenterology, Department of Pediatrics and Adolescent Medicine, Comprehensive Center for Pediatrics, Medical University of Vienna, Vienna, Austria; ^4^ Christian Doppler Laboratory for Molecular Stress Research in Peritoneal Dialysis, Department of Pediatrics and Adolescent Medicine, Medical University of Vienna, Vienna, Austria; ^5^ Institute of Cancer Research, Department of Medicine I, Medical University of Vienna and Comprehensive Cancer Center, Vienna, Austria

**Keywords:** lung transplantation, induction therapy, alemtuzumab, ATG (anti-thymocyte globulin), serum proteomics, mass spectrometry

## Abstract

**Introduction:**

Induction therapy is widely used in lung transplantation to control the host alloresponse, reducing acute cellular rejection and improving graft survival. Despite its use, data on the biological effects of different induction agents remain limited.

**Methods:**

This study examines serum proteomics profiles in lung transplant patients receiving alemtuzumab, anti-thymocyte globulin (ATG), or no induction therapy. Adult lung transplant recipients who underwent transplantation between 2007 and 2013 at the Medical University of Vienna were included. Using mass spectrometry (MS), serum samples were examined before transplantation (T1) and 12 months post-transplant (T2).

**Results:**

Among 102 patients (50 alemtuzumab, 34 ATG, 18 no induction), we identified significantly differentially expressed proteins over time and between groups at T2. In the alemtuzumab group, 40 proteins were differentially expressed (3 upregulated, 37 downregulated), in ATG, 22 proteins (3 upregulated, 19 downregulated), and none in the no-induction group. At T2, two proteins (fibulin-1 and fetuin-B) were downregulated between alemtuzumab and no induction, with no significant differences between alemtuzumab and ATG or ATG and no induction.

**Discussion:**

Our findings suggest alemtuzumab may have a stronger effect on circulating proteome. Further studies are warranted to elucidate the underlying mechanisms and explore potential clinical implications.

## 1 Introduction

Lung transplantation is a life-saving procedure for patients with end-stage lung diseases, such as chronic obstructive pulmonary disease (COPD), idiopathic pulmonary fibrosis, and cystic fibrosis. Despite the considerable advancements, lung transplantation is associated with significant challenges, particularly in managing acute and chronic rejection, infections, and complications arising from long-term immunosuppression (1, 2). Effective immunosuppressive strategies are crucial to enhancing graft survival and patient outcomes, with induction therapy playing a pivotal role in this context.

Induction immunosuppression is routinely used (3) to reduce the cumulative amount of maintenance immunosuppression and to lower the rate of acute cellular rejection (ACR) by targeting T-cell mediated responses, thereby reducing the rate of chronic rejection (CLAD). The main induction agents used worldwide are alemtuzumab, a monoclonal antibody targeting CD52 antigen (4–6), anti-thymocyte globulin (ATG), a polyclonal antibody preparation with multiple targets, and basiliximab, an antagonist of the IL-2 receptor (7–13).

Despite the clinical success of these induction therapies, the underlying molecular mechanisms and their impact on the proteomic landscape of transplant recipients remain areas of active investigation. Proteomics, offers a powerful approach to understanding the complex biological processes involved in transplantation. Previous research has underscored the potential of serum proteomics to identify biomarkers for various clinical outcomes in transplantation (14, 15). Additionally, proteomic analyses have been instrumental in identifying novel biomarkers for monitoring post-transplantation complications and treatment efficacy (16–19).

In this study, we aimed to investigate the serum proteomics profiles of lung transplant patients receiving different induction therapies: alemtuzumab, ATG, and no induction therapy. Our hypothesis is that induction agents do not only have a short-term effect on T-cell alloresponse but their depleting effect might mediate long-term changes in lung transplant recipients. Using mass spectrometry techniques and the LIMMA platform for data analysis, we aimed to identify significantly differentially expressed proteins within each group over time and between different groups. By integrating Gene Ontology (GO), Molecular Function (MF), Biological Process (BP), and KEGG pathway analyses, we sought to uncover the biological processes and pathways affected by these induction therapies.

## 2 Materials and methods

### 2.1 Study population

This study included adult lung transplant recipients who underwent lung transplantation between 2007 and 2013 at the Department for Thoracic Surgery at the Medical University of Vienna. In this time period, 666 lung transplantation were performed in our institution. Patients receiving either alemtuzumab, ATG or no induction were included in the study. Exclusion criteria were retransplantation or multi-organ transplantation, pediatric patients, single lung transplantation or a follow-up time shorter than 12 months. In addition, the biobank at our institution was not granular during this time period, so only patients who had provided samples both at baseline and 12 months were included in the analysis. Patients received immunosuppression protocols, as described elsewhere (5). Briefly, until 2009 we used ATG induction in cystic fibrosis (CF) and idiopathic pulmonary hypertension (IPAH) patients, all other recipients received no induction. After 2009, alemtuzumab was implemented as standard treatment for all recipients with exception of patients with graft versus host disease (GvHD) after bone marrow transplantation and patients colonized by species of *Burkholderia* or resistant *Mycobacterium abscessus* prior to lung transplantation, who did not receive any induction therapy. Severe intraoperative bleeding or the use of an extracorporeal life support (ECLS) device as bridge to lung transplantation represented a contraindication for the use of ATG, in order to minimize bleeding risk in the post-operative period. ATG was intravenously administrated at a dose of 2 mg/kg on postoperative day (POD) 0, 1, 2, 3 and 4, while alemtuzumab was given as a single dose of 30 mg after arrival on the intensive care unit (ICU). Maintenance immunosuppression regimen was based on triple-drug treatment combination of tacrolimus, mycophenolate mofetil (MMF) and steroids. Tacrolimus was started immediately after transplantation with target blood levels depending on the type of induction therapy. Target blood levels are described in **Supplementary Table 1**. MMF was started based on leukocyte count and clinical course, with a standard dose of 2-3g/day. In patients receiving alemtuzumab, MMF was usually started one year after transplantation. Biopsies were classified according to ISHLT criteria (20). ACR grade A2 and LB grade B2 or higher were treated with a pulse of steroids for 3 days with consecutive dose tapering. In case of inadequate clinical response, ATG (2 mg/kg) was administered for 5 days. Until 2014, measurement of donor-specific antibodies was not available at our center. Diagnosis of CLAD was established by two independent physicians according to the consensus report of the ISHLT (21). The study complied with the Declaration of Helsinki and had been approved by the Institutional Ethical Committee of the Medical University of Vienna (ECS 1891/2016).

### 2.2 Serum sample collection

Serum samples were collected prospectively at two time points: at day of transplantation before the procedure (T1) and one year post-transplantation (T2). Each patient had paired samples, meaning the same patients were followed and sampled again one year after transplantation. Venous blood was collected from each patient in VACUETTE^®^ CAT serum clot activator tubes with a silica based coagulating agent. Samples were allowed to clot at room temperature for approximately 2 hours. After clot formation, the samples were centrifuged at 1500 g for 10 minutes. The serum was then aliquoted and stored at -80°C within 30 minutes of collection until proteomic analysis. Each serum sample underwent only one freeze/thaw cycle before analysis.

### 2.3 Proteomic analysis

Serum samples were thawed on ice and processed with Top 12 Abundant Protein Depletion Spin Columns (Pierce Biotechnology, Rockford, lL, USA) per manufacturer’s instructions (10 µl input material). Filtrates were precipitated using methanol/dichloromethane and digested with trypsin as previously described (22). The precipitated proteins were dissolved in 0.1% Rapigest (Waters, Vienna, Austria), reconstituted in 50 mM triethylammonium bicarbonate, and protein concentration was determined using a DS-11 series spectrophotometers/fluorometers (DeNovix, Wilmington, DE, USA). All steps were carried out using Protein LoBind tubes (Eppendorf, Hamburg, Germany). Proteins were reduced with 5 mM DTT for 30 min at 60°C, and alkylated for 30 min with 15 mM IAA in the dark, and then digested for 16 h at 37°C using a trypsin ratio of 1:50. Digestion was stopped by acidification with trifluoroacetic acid (TFA). Following injection of 1 µg in 20 µl onto the trapping column (Acclaim C18 trap column, 300 μm inner diameter × 5 mm), peptides were separated by nano-reverse-phase chromatography (Acclaim C18, 75 μm inner diameter × 500 mm) using an UltiMate nano RSLC HPLC system (Thermo Fisher, Germering, Germany), which included the autosampler, column switching unit, nano and loading pump, and UV detector. Both trap and separation columns were operated at 60°C, with UV peptide detection at 214 nm serving as quality control for HPLC separation. Samples were loaded onto the trap column using 0.1% TFA at 30 μL/min and precooled to 3°C. A user-defined injection program was used for sample injection, including additional injector and trap column washes. Briefly, after sample loading, the gradient program began at 5% mobile phase B (80% acetonitrile in water, 0.1% formic acid (FA)) and increased linearly to 20% MPB over 120 minutes. During the next 30 minutes, MPB was raised to 25% and in the third step, it was raised from 25% to 40% in 30 minutes, followed by washing and equilibration at the end. The flow rate was set to 0.8 µl/min from 0 to 12 minutes and from 215 to 225 minutes; otherwise, it was set to 0.6 µl/min. Each sample injection was followed by two blank runs with injections of 2,2,2-trifluoroethanol to remove potential sample remnants and prevent carryover. MS analysis was performed using the Q-Exactive Plus mass spectrometer (Thermo Fisher Scientific) employing the “top 20” method for MS/MS experiments. This method selects the 20 most intense ions from the MS scan for tandem MS (MS/MS) analysis. Single-charged ions were excluded from fragmentation, and detected ions were excluded from further fragmentation for 2 minutes after initial MS/MS fragmentation. MS resolution was set to 70,000 with an AGC target of 3E6 ions, while MS/MS resolution was set to 35,000 with an AGC target of 1E5 ions. Fragmentation was performed using higher-energy collisional dissociation (HCD) with a normalized collision energy of 30 eV. Data analysis, including database search and label-free quantification, was performed using Proteome Discoverer (v2.4.0.305, Thermo Fisher). SequestHT was used as search engine and the following parameters were chosen: database - Homo sapiens (fasta file downloaded from UniProt/SwissProt on 2 February 2023, comprising 20,389 proteins and 42,382 isoforms); enzyme - trypsin; max. missed cleavage sites - 2; static modifications: carbamidomethyl (C); dynamic modifications: oxidation (M), deamidation (N, Q), acetyl (protein N-terminus), Met-loss (M) and Met-loss + acetyl (M); precursor mass tolerance - 10 ppm; fragment mass tolerance - 0.02 Da. Precursor ion quantification was done using the Minora Feature Detector node. Retention time alignment was performed with a maximum RT shift of 10 min and a mass tolerance of 10ppm. For feature linking the RT and mass tolerance were set to 0 with a minimum S/N threshold of 5. Intensity-based label-free quantification was performed using unique peptides only. Normalization in Proteome Discoverer was based on total peptide amount and scaling mode on “all average”.

### 2.4 Bioinformatics and statistical analysis

Categorical variables were reported as absolute and relative frequencies (%), continuous variables as median (interquartile range, IQR) or mean (± standard deviation). Chi-square tests, Fisher exact tests, Mann-Whitney U-tests, or ANOVA were used to compare variables as applicable. Proteomic data analysis was performed using the LIMMA platform using log2-transformed normalized and scaled data exported from the Proteome Discoverer software. For LIMMA analysis, only proteins with at least three abundances per group were used. Batch effects were assessed by monitoring regular injections of system suitability controls (Pierce HeLa Protein Digest Standard, Thermo Fisher Scientific) and mitigated by paired samples from each patient as a blocking variable in the linear mixed model analysis (treated as random effect via duplicateCorrelation in LIMMA). Comparisons of protein abundance were made between T2 and T1 within each group, and between different groups at T2. Additionally, inter-group comparisons at T1 were conducted to assess baseline proteome similarities, and these results are included in the **Supplementary Material** (**Supplementary Tables 2**, **3**). Differentially expressed proteins were identified using paired comparisons: alemtuzumab T2 vs. alemtuzumab T1, ATG T2 vs. ATG T1, and no-induction T2 vs. no-induction T1. Inter-group comparisons were also made at T2: alemtuzumab T2 vs. no-induction T2 and alemtuzumab T2 vs. ATG T2. Significant dysregulation was considered with an adjusted p-value < 0.05 using the Benjamini-Hochberg correction. The significantly differentially expressed proteins were further analyzed using the gProfiler web tool for Gene Ontology (GO), Molecular Function (MF), Biological Process (BP), Cellular Component (CC), and KEGG pathway analysis. Data was analyzed using LIMMA and the g:Profiler online tool, and graphics were designed with GraphPad Prism. Intensity-based label-free quantification was performed using unique peptides only. Normalization was based on total peptide amount and scaling mode on all average in Proteome Discoverer, followed by log-transformation of the normalized values and statistical analysis using the LIMMA package in R.

## 3 Results

### 3.1 Demographics of the study cohort

**Figure 1:**
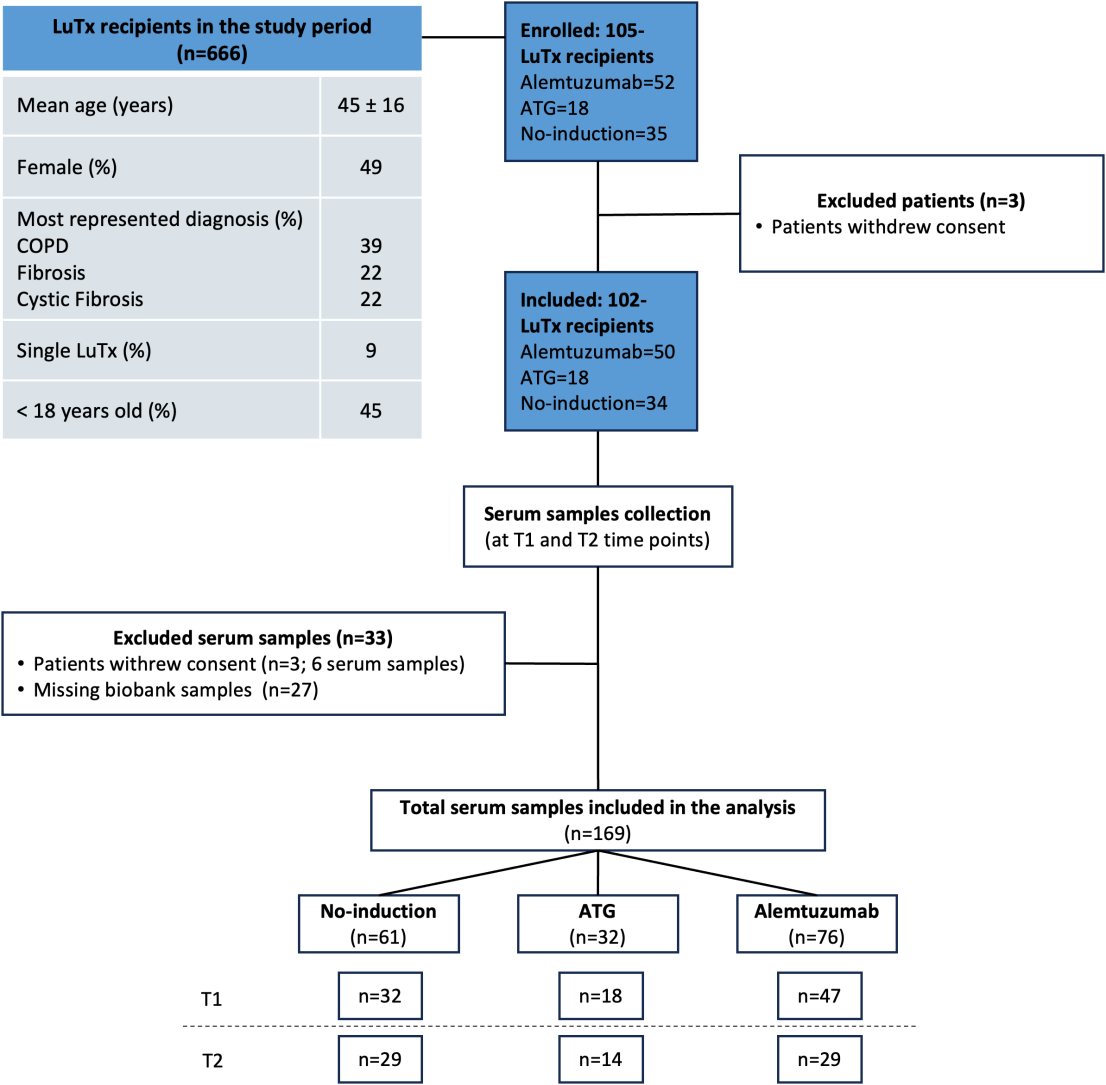
CONSORT chart. A total of 102 lung transplantation recipients were included in the study (alemtuzumab=50, ATG=18, and no-induction therapy=34). Serum samples were collected at two time points: pre-transplantation (T1), and one year post-transplantation (T2). Thirty-three serum samples were excluded: three patients withdrew consent (n = 6 samples), and twenty-seven samples were unavailable from the biobank (due to lack of stored serum sample or suboptimal storage conditions). A total of 169 serum samples were included in the final analysis.

In the study period, 666 patients received lung transplantation. Mean age was 45 ± 16 year and 49% were female. The most represented diagnosis were COPD, fibrosis and CF in 39%, 22% and 22% of patients, respectively. Single-lung transplantation was performed in 60 (9%) patients. Forty-one patients were younger than 18 years old. In 45% (n=297) of the patients, long-term follow-up was carried out in peripheral centers or abroad, so that no regular biosampling was performed. One-hundred one patients consented to the biosampling before transplantation but withdrew their consent within the first 12 months post-transplant. A total of 102 lung transplant recipients were included in the analysis. Mean follow-up time was 110 months (SD 56 months). Among these, 50 patients received alemtuzumab induction therapy, 34 received anti-thymocyte globulin (ATG) induction therapy, and 18 received no induction therapy. An overview of the study design is provided in the CONSORT chart (**Figure 1**), and the full demographics are shown in **Table 1**. Baseline characteristics of the recipients were not different among the study groups. Incidence of ACR and lymphocytic bronchiolitis (LB) within the first 12 months after transplantation was significantly higher in the no induction and ATG group. Freedom from CLAD at 1 year was: 97% in no induction group, 94% in ATG group and 98% in alemtuzumab group. Freedom from CLAD at 5 years was: 33% in no induction group, 77% in ATG group and 70% in alemtuzumab group.

**Table 1:** Patient demographics and clinical characteristics by induction therapy in this study.

Characteristics		No induction (n=34)	ATG (n=18)	Alemtuzumab (n=50)	p-value
Age (mean ± SD)		54 ± 9	49 ± 12	50 ± 12	0.131
Female (n, %)		20 (63%)	11 (61%)	23 (49%)	0.505
Diagnosis (n, %)	Obstructive	27 (84%)	13 (72%)	15 (32%)	0.308
	Fibrotic	2 (6%)	2 (11%)	13 (28%)	
	Vascular	1 (4%)	0	2 (4%)	
	Infectious	2 (6%)	3 (17%)	17 (36%)	
High-risk CMV mismatch (n, %)		6 (19%)	4 (22%)	11 (23%)	0.971
Cumulative A score (median, IQR)		0.13 (0.07 - 0.31)	0 (0 - 0.2)	0 (0 – 0.03)	<0.001
Cumulative B score (median, IQR)		0.7 (0.5 – 1)	0.55 (0.3 – 0.88)	0.32 (0.13 – 0.50)	<0.001
High grade ACR ≥ A2 (n, %)		10 (31%)	3 (17%)	0	0.003
High grade LB ≥ B2 (n, %)		17 (53%)	8 (44%)	9 (19%)	0.036
CLAD within follow-up time		27 (84%)	6 (33%)	21 (45%)	0.006
CLAD phenotype	BOS	18 (67%)	5 (83%)	13 (62%)	<0.001
	RAS	6 (22%)	0	2 (10%)	
	Mixed	3 (11%)	1 (17%)	6 (29%)	
CLAD Grade	1	10 (37%)	3 (50%)	4 (19%)	0.064
	2	8 (30%)	1 (17%)	4 (19%)	
	3	9 (33%)	2 (33%)	10 (48%)	
	4	0	0	3 (3%)	

### 3.2 Protein abundance and identification

A visual representation of the distribution of log2-transformed protein abundances across different groups and time points is shown in **Supplementary Figure 1**. The overall distribution of protein abundances is similar across the groups and time points, indicating consistent sample quality and preparation across the study. The overall overlap of protein identifications across the samples is depicted in **Supplementary Figures 1B–D**. The majority of proteins were consistently identified across multiple samples, ensuring robust and reliable data for subsequent analyses.

Principal Component Analysis (PCA) was performed to visualize the variation in proteomic profiles among the different groups and time points (**Supplementary Figure 2**). Within the alemtuzumab and ATG groups, samples from T1 and T2 showed partial separation, suggesting time-dependent changes in serum proteomes. In the alemtuzumab group, although there is some overlap between time points, a distinct cluster consisting exclusively of T2 samples is evident in the top center of the plot. In contrast, the ATG group displays a more pronounced separation, with T1 and T2 samples clustering predominantly on the left and right sides of the plot, respectively. In the No-induction group, samples from T1 and T2 exhibit extensive overlap (**Supplementary Figures 2A–C**). When focusing solely on T2 samples across all groups, despite some intermixing, most No-induction samples grouped separately from the alemtuzumab and ATG samples (**Supplementary Figure 2D**). In the combined PCA (all time points and groups), although overlapping is evident, the majority of alemtuzumab T2 samples nevertheless co‐localized toward the bottom right of the plot (**Supplementary Figure 2E**).

### 3.3 Proteomic changes over time within induction therapy groups

#### 3.3.1 Alemtuzumab

In the comparison between alemtuzumab T2 and alemtuzumab T1, a total of 40 proteins were significantly differentially expressed. Among these, 3 proteins were upregulated and 37 were downregulated (**Table 2**).

**Table 2:** List of differentially expressed proteins in alemtuzumab T2 vs. alemtuzumab T1.

Alemtuzumab_T2_vs._Alemtuzumab_T1				
Upregulated proteins				
Gene	Description	p-value	FDR	log2FC
APOA1	Apolipoprotein A-I	7.70E-05	0.00274	1.39045564
ALB	Albumin	0.00259	0.03638	0.99161573
SPP2	Secreted Phosphoprotein 24	0.00341	0.03999	1.20914036
Downregulated proteins				
IGHA1	Immunoglobulin heavy constant alpha 1 OS=Homo sapiens OX=9606 GN=IGHA1 PE=1 SV=2	2.9555E-08	1.0758E-05	-1.9097055
TMSB4X	Thymosin beta-4 OS=Homo sapiens OX=9606 GN=TMSB4X PE=1 SV=2	7.1156E-07	0.0001295	-1.7256853
TPM4	Tropomyosin alpha-4 chain OS=Homo sapiens OX=9606 GN=TPM4 PE=1 SV=3	1.1508E-06	0.00013963	-1.8966776
ICAM1	Intercellular adhesion molecule 1 OS=Homo sapiens OX=9606 GN=ICAM1 PE=1 SV=2	1.8117E-06	0.00016487	-1.6071854
CFHR5	Complement factor H-related protein 5 OS=Homo sapiens OX=9606 GN=CFHR5 PE=1 SV=1	25898E-06	0.00018854	-1.1431494
IGHG2	Immunoglobulin heavy constant gamma 2 OS=Homo sapiens OX=9606 GN=IGHG2 PE=1 SV=2	1.2068E-05	0.00073215	-1.8004895
A1BG	Alpha-1B-glycoprotein OS=Homo sapiens OX=9606 GN=A1BG PE=1 SV=4	4.0576E-05	0.00210993	-1.0106048
IGHA2	Immunoglobulin heavy constant alpha 2 OS=Homo sapiens OX=9606 GN=IGHA2 PE=1 SV=4	8.2767E-05	0.00273891	-1.7330179
SAA2	Serum amyloid A-2 protein OS=Homo sapiens OX=9606 GN=SAA2 PE=1 SV=1	6.8071E-05	0.00273891	-1.8731789
ECM1	Extracellular matrix protein 1 OS=Homo sapiens OX=9606 GN=ECM1 PE=1 SV=2	9.0294E-05	0.00273891	-1.0401136
CADM1	Cell adhesion molecule 1 OS=Homo sapiens OX=9606 GN=CADM1 PE=1 SV=2	8.4751E-05	0.00273891	-1.9067821
IGHG4	Immunoglobulin heavy constant gamma 4 OS=Homo sapiens OX=9606 GN=IGHG4 PE=1 SV=1	0.00013452	0.00376654	-1.8351159
FETUB	Fetuin-B OS=Homo sapiens OX=9606 GN=FETUB PE=1 SV=2	0.00033897	0.00881312	-0.9707794
CFI	Complement factor I OS=Homo sapiens OX=9606 GN=CFI PE=1 SV=2	0.00046789	0.01135421	-0.7334874
IGKC	Immunoglobulin kappa constant OS=Homo sapiens OX=9606 GN=IGKC PE=1 SV=2	0.00068949	0.01476326	-1.0930093
FGG	Fibrinogen gamma chain OS=Homo sapiens OX=9606 GN=FGG PE=1 SV=3	0.00068326	0.01476326	-1.6703199
FBLN1	Fibulin-1 OS=Homo sapiens OX=9606 GN=FBLN1 PE=1 SV=4	0.00090019	0.01724578	-1.9014911
LTBP1	Latent-transforming growth factor beta-binding protein 1 OS=Homo sapiens OX=9606 GN=LTBP1 PE=1 SV=4	0.00089246	0.01724578	-1.1619318
PFN1	Profilin-1 OS=Homo sapiens OX=9606 GN=PFN1 PE=1 SV=2	0.00126932	0.02310154	-1.3701953
PPBP	Platelet basic protein OS=Homo sapiens OX=9606 GN=PPBP PE=1 SV=3	0.00143684	0.02490521	-0.8562653
IGFBP2	Insulin-like growth factor-binding protein 2 OS=Homo sapiens OX=9606 GN=IGFBP2 PE=1 SV=2	0.00197049	0.03260263	-0.9902271
PDLIM1	PDZ and LIM domain protein 1 OS=Homo sapiens OX=9606 GN=PDLIM1 PE=1 SV=4	0.00233066	0.03534829	-1.7364387
PPIA	Peptidyl-prolyl cis-trans isomerase A OS=Homo sapiens OX=9606 GN=PPIA PE=1 SV=2	0.00231497	0.03534829	-1.1860468
F5	Coagulation factor V OS=Homo sapiens OX=9606 GN=F5 PE=1 SV=4	0.00259837	0.03637723	-0.7488396
TPM3	Isoform 2 of Tropomyosin alpha-3 chain OS=Homo sapiens OX=9606 GN=TPM3	0.00282663	0.03810719	-1.4113404
SSC5D	Soluble scavenger receptor cysteine-rich domain-containing protein SSC5D OS=Homo sapiens OX=9606 GN=SSC5D PE=1 SV=3	0.00324524	0.03998783	-1.7602801
CFL1	Cofilin-1 OS=Homo sapiens OX=9606 GN=CFL1 PE=1 SV=3	0.00330466	0.03998783	-1.3299549
PCSK9	Proprotein convertase subtilisin/kexin type 9 OS=Homo sapiens OX=9606 GN=PCSK9 PE=1 SV=3	0.00312991	0.03998783	-1.250213
F2	Prothrombin OS=Homo sapiens OX=9606 GN=F2 PE=1 SV=2	0.00352308	0.04007507	-0.5570216
CALU	Calumenin OS=Homo sapiens OX=9606 GN=CALU PE=1 SV=2	0.00372881	0.04100497	-3.2572384
VTN	Vitronectin OS=Homo sapiens OX=9606 GN=VTN PE=1 SV=1	0.00383013	0.04100497	-0.3949694
PRSS1	Serine protease 1 OS=Homo sapiens OX=9606 GN=PRSS1 PE=1 SV=1	0.00404814	0.04210067	-1.9317487
MBL2	Mannose-binding protein C OS=Homo sapiens OX=9606 GN=MBL2 PE=1 SV=2	0.00464877	0.04574791	-0.8578669
IGFBP3	Insulin-like growth factor-binding protein 3 OS=Homo sapiens OX=9606 GN=IGFBP3 PE=1 SV=2	0.0046502	0.04574791	-0.7406332
JCHAIN	Immunoglobulin J chain OS=Homo sapiens OX=9606 GN=JCHAIN PE=1 SV=4	0.00520963	0.04868268	-1.3257171
A1BG	Isoform 2 of Alpha-1B-glycoprotein OS=Homo sapiens OX=9606 GN=A1BG	0.005216	0.04868268	-1.6908796
ICAM2	Intercellular adhesion molecule 2 OS=Homo sapiens OX=9606 GN=ICAM2 PE=1 SV=2	0.0054015	0.04915368	-1.2599918

The table presents the list of significantly differentially expressed proteins between the alemtuzumab T2 and alemtuzumab T1 time points with adjusted FDR below 0.05. Proteins are categorized into upregulated and downregulated groups. For each protein, the table includes the gene symbol, full description, log2 fold change (log2FC), p-value, and false discovery rate (FDR). FDR was calculated using the Benjamini-Hochberg method, with a significance threshold set at FDR < 0.05.

The upregulated proteins included Apolipoprotein A-I (apoA-I), albumin, and Secreted Phosphoprotein 24 (SPP2). Among the downregulated proteins, most proteins have a direct function within the immune system. For instance, several subunits of immunoglobulin Heavy Constant, immunoglobulin kappa constant, immunoglobulin J chain were downregulated. Similar downregulation was observed for Immunoglobulin Heavy Constant Gamma 4 (IGHG4). Levels of both Intercellular Adhesion Molecule 1 (ICAM1) and 2 (ICAM2), were reduced at T2 compared to baseline, suggesting reduced leukocyte adhesion and migration. Both complement factor H-related protein 5 (CFHR5) and complement factor I were downregulated, suggesting suppression of complement activity. Similarly, levels of Complement Component 3 (C3), were decreased. The results of Gene Ontology analysis for this comparison, including molecular function, biological process, and KEGG pathway analysis, are presented in **Supplementary Figure 3**.

#### 3.3.2 ATG

A total of 22 proteins were significantly differentially expressed in the ATG group at T2 compared to T1. Among these, 3 proteins were upregulated and 19 were downregulated (**Table 3**).

**Table 3:** List of differentially expressed proteins in ATG T2 vs. ATG T1.

ATG T2 vs. ATG T1				
Upregulated proteins				
Gene	Description	p-value	FDR	log2FC
IGHM	Immunoglobulin Heavy Constant Mu	0.00228	0.04470	1.8136912
ALB	Albumin	0.00236	0.04470	1.50643858
MCAM	Melanoma Cell Adhesion Molecule	0.00300	0.04959	1.84775383
Downregulated proteins				
C1QB	Complement C1q Subcomponent Subunit B	1.26E-05	0.00460	-1.7967639
ITIH1	Inter-Alpha-Trypsin Inhibitor Heavy Chain H1	6.71E-05	0.01221	-1.6254809
PZP	Pregnancy Zone Protein	0.00016	0.01636	-2.4712747
LYVE1	Lymphatic Vessel Endothelial Hyaluronan Receptor 1	0.00018	0.01636	-20490143
C1QA	Complement C1q Subcomponent Subunit A	0.00024	0.01722	-1.8539079
LYZ	Lysozyme	0.00028	0.01722	-2.1365697
CFHR2	Complement Factor H-Related Protein 2	0.00038	0.01969	-1.7101512
AGT	Angiotensinogen	0.00065	0.02649	-1.5624898
VTN	Vitronectin	0.00074	0.02649	-0.925754
LRG1	Leucine-Rich Alpha-2-Glycoprotein 1	0.00077	0.02649	-1.3337751
TMSB4X	Thymosin Beta-4 X-Linked	0.00088	0.02649	-0.696414
C3	Complement Component 3	0.00089	0.02649	-1.7073089
ECM1	Extracellular Matrix Protein 1	0.00095	0.02649	-1.3071993
SERPINA5	Alpha-1-Antitrypsin	0.00106	0.02756	-1.9835459
F2	Coagulation Factor II (Thrombin)	0.00114	0.02764	-0.9334852
CFH	Complement Factor H	0.00136	0.03091	-0.9839555
AZGP1	Zinc-Alpha-2-Glycoprotein	0.00148	0.03159	-1.3551162
C1R	Complement C1r Subcomponent	0.00246	0.04470	-0.9889098
SERPINF2	Alpha-2 Antiplasmin	0.00285	0.04943	-0.9495036

The table presents the list of significantly differentially expressed proteins between the ATG T2 and ATG T1 time points with adjusted p-values below 0.05. Proteins are classified as upregulated or downregulated. For each protein, the table includes the gene symbol, full description, log2 fold change (log2FC), p-value, and false discovery rate (FDR). FDR was calculated using the Benjamini-Hochberg method, with a significance threshold set at FDR < 0.05.

The upregulated proteins included Immunoglobulin Heavy Constant Mu (IGHM), which plays a crucial role in the immune response by mediating the primary antibody response, albumin (ALB), and Melanoma cell adhesion molecule (MCAM, also known as CD146), which is involved in cell adhesion, signaling, and immune responses.

Among the downregulated proteins, a significant subset showed direct associations with immune responses. For instance, several complement factors, namely C1QB, C1QA, CFHR2, C3, CFH and C1R were all downregulated, suggesting a suppressive effect of ATG on circulating complement factors. Conversely, acute inflammatory proteins, such as inter-alpha-trypsin inhibitor heavy chain H1 (ITIH1), was also downregulated at T2. Lymphatic vessel endothelial hyaluronan receptor 1 (LYVE1), a type I integral membrane glycoprotein mainly located in the lymphatic vessels and responsible for their integrity was downregulated in the ATG group at T2. Angiotensinogen, Vitronectin, Leucin-Rich Alpha-2 Glycoprotein 1, Thymosin Beta-4 X-Linked, Extracellular Matrix Protein 1, Alpha-1-Antitrypsin, Coagulation Factor II, Complement Factor H, Zinc-Alpha-2-Glycoprotein and Alpha-2 Antiplasmin were all downregulated at T2 compared to baseline in the ATG group. **Supplementary Figure 4** summarizes the Gene Ontology analysis results for this comparison, highlighting molecular function, biological process, and KEGG pathway findings.

#### 3.3.3 No-induction

No significantly differentially expressed proteins were found in the no-induction at T2 compared to baseline.

### 3.4 Comparative serum proteomics between groups at baseline

At T1, comparative analysis of serum proteomes revealed differences between the induction therapy groups (**Supplementary Tables 2**, **3**). These findings highlight a degree of biological heterogeneity across the groups prior to the administration of induction therapy, which is reflected in their baseline serum proteomic profiles.

### 3.5 Comparative serum proteomics between groups 12 months post-transplant

No proteins were significantly upregulated when comparing the alemtuzumab and no-induction groups at T2. Two proteins were significantly downregulated: Fibulin-1 and Fetuin-B (**Table 4**).

**Table 4:** List of differentially expressed proteins in alemtuzumab T2 vs. no-induction T2.

Alemtuzumab T2 vs. No-induction T2				
Downregulated proteins				
Gene	Description	p-value	FDR	log2FC
FBLN1	Fibulin-1	9.43E-06	0.00343	-2.8752748
FETUB	Fetuin B	4.54E-05	0.00826	-1.2519159

The table presents the list of significantly downregulated proteins between the alemtuzumab T2 and no-induction T2 time points. For each protein, the table includes the gene symbol, full description, log2 fold change (log2FC), p-value, and false discovery rate (FDR). FDR was calculated using the Benjamini-Hochberg method, with a significance threshold set at FDR < 0.05.

**Figure 2:**
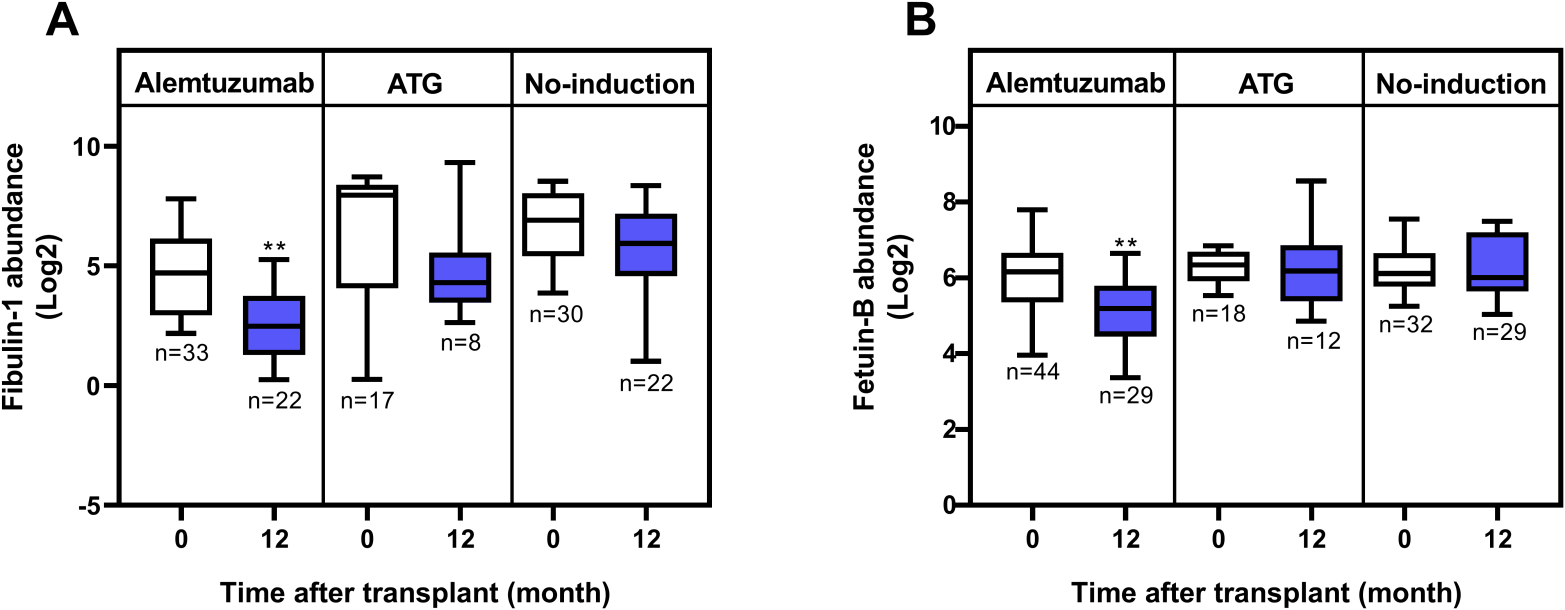
Serum levels of Fibulin-1 and Fetuin-B in lung transplant recipients. **(A)** Fibulin-1 levels pre- and 12-months post-transplant across induction therapies. While Fibulin-1 levels decreased over time in all groups, statistical significance was only reached in the alemtuzumab group (p = 0.0015 for alemtuzumab; p = 0.1943 for ATG; and p = 0.1357 for no-induction). **(B)** Fetuin-B levels pre- and 12-months post-transplant across induction therapies. Significant downregulation was observed in the alemtuzumab group (p = 0.0021), but not in the ATG (p = 0.6539) or no-induction (p = 0.9457) groups. Statistical analysis was performed using the Mann–Whitney U test. Boxes represent the median and interquartile range; whiskers show 10th–90th percentiles. p-value significance is indicated by asterisks: * = p < 0.05; ** = p < 0.01.

Fibulin-1, which is involved in extracellular matrix organization and tissue remodeling, was already significantly lower in the alemtuzumab group compared to both the no-induction and ATG groups at baseline. Its serum levels decreased over time in all three groups; however, the reduction was only statistically significant in the alemtuzumab group (**Figure 2A**). In contrast, Fetuin-B exhibited a distinct and specific downregulation in the alemtuzumab group one year after transplantation, while its serum levels in the ATG and no-induction groups remained unchanged (**Figure 2B**). No significantly differentially expressed proteins were found when comparing both alemtuzumab and ATG as well as ATG and no-induction at T2.

## 4 Discussion

This study provides a comprehensive proteomic analysis of lung transplant recipients treated with distinct induction therapies—alemtuzumab, ATG, and no-induction. By analyzing serum samples, we identified key differences in the proteomic landscape at one year post-transplantation, shedding light on the distinct immunomodulatory effects of these therapies.

Within the alemtuzumab group, comparison of serum samples one year post-transplantation to pre-transplantation revealed a more extensive dysregulation of proteins over time (**Table 2**, **Supplementary Figure 7**). These proteins were primarily associated with immune regulation, inflammation, and extracellular matrix activity, suggesting a shift toward reduced immune activation and inflammation. Upregulation of negative acute-phase proteins, apoA-I and albumin, known for its anti-inflammatory and antioxidative properties, aligns with this observed immunosuppressive phenotype (23–34). Conversely, the downregulation of immune-related proteins such as immunoglobulin subunits, complement factors, and adhesion molecules (e.g., ICAM1/2), as well as positive acute-phase proteins such as SAA2, A1BG, and FGG, highlights the suppression of leukocyte recruitment, complement activation, and inflammatory signaling pathways (35–37). These findings illustrate that alemtuzumab effectively modulates key proteins involved in immune response, inflammation, and extracellular matrix organization. The upregulation of protective proteins like apoA-I, coupled with the downregulation of several immune and inflammation related proteins, may suggest a role for alemtuzumab in creating a favorable immunosuppressive environment post-transplantation, however, this finding needs to be confirmed in future studies.

In contrast, ATG treatment led to fewer differentially expressed proteins within the group, one year post-transplantation compared to pre-transplantation, with evidence of both immune suppression and residual activation (**Table 3** and **Supplementary Figure 8**). While complement components were downregulated, upregulation of immunoglobulin components such as IGHM, and MCAM, a protein involved in cell adhesion, signaling, and immune responses, points toward ongoing immune activation (23, 38). On the other hand, downregulation of acute-phase proteins, such as ITIH1, which has been shown in kidney transplant patients to decrease in plasma concentrations during acute inflammation, was observed at T2 (39).

When comparing the groups one year post-transplantation, both Fibulin-1 and Fetuin-B levels were significantly lower in the alemtuzumab group compared to the No-induction group (**Table 4** and **Supplementary Figure 9**). Although a similar downward trend of Fibulin-1 was also observed over time in the ATG and No-induction groups, it did not reach statistical significance (**Figure 2A**). Notably, serum Fibulin-1 levels were already significantly lower at T1 in the alemtuzumab group compared to that of ATG or no-induction group (**Supplementary Tables 2**, **3**). This baseline difference suggests potential pre-existing heterogeneity among patient groups. Fibulin-1 has been shown to promote tumor survival via activation of anti-apoptotic Notch signaling and is associated with increased immune cell infiltration in hepatocellular carcinoma (HCC), highlighting its possible involvement in tissue remodeling and immune regulation (40). In pulmonary fibrosis, fibulin-1C isoform enhances airway smooth muscle and fibroblast adhesion, proliferation, and extracellular matrix deposition (41). Further studies are needed to explore its potential role in post-transplantation tissue remodeling and immunological changes.

On the other hand, the specific downregulation of Fetuin-B was only observed in the alemtuzumab group over time (**Figure 2B**). Fetuin-B, a liver-derived serum glycoprotein, is involved in metabolic regulation and acute-phase responses. Previous studies have shown that reduced Fetuin-B levels are associated with improved metabolic profiles and reduced inflammation (42). Moreover, recent studies have demonstrated that Fetuin-B induces a pro-inflammatory response and is associated with cytokine/chemokine signaling pathways in adipocytes and plasma (43, 44). In lung disease, Fetuin-B has emerged as a potential biomarker, with its levels progressively increasing in COPD patients and correlating negatively with lung function (45). Additionally, *in vitro* studies have demonstrated that Fetuin-B can activate the NF-κB signaling pathway, leading to upregulation of pro-inflammatory markers like TNF-α, VEGF, and IBA-1 in microglial cells, highlighting its potential role in inflammation and immune responses (46). Notably, Fetuin-B levels remained unchanged in both ATG and no-induction groups, emphasizing its potential specificity to alemtuzumab’s immunomodulatory effects (**Figure 2B**). The observed decrease raises the possibility that Fetuin-B could be a novel mediator linking alemtuzumab to the suppression of inflammatory pathways, a hypothesis that warrants further mechanistic investigation.

Limitations of this mechanistic study include an unbalanced sample size between the different groups and the lack of randomization between different immunosuppressive strategies. While ATG and alemtuzumab appear statistically similar, the lack of difference between ATG and no induction at 12 months post-transplantation may reflect limited statistical power. The ATG group included only 14 patients at T2, which may reduce the ability to detect meaningful differences. Moreover, the more extensive differentially expressed proteins (DEPs) in the alemtuzumab group compared to ATG may partly reflect technical variability, as the number of identified proteins varied more widely in the larger alemtuzumab group. Whether a larger sample size or later time points would reveal differences remains unclear. Additional time points, both at 6 months and beyond 12 months, could have provided further valuable insights into the dynamics of proteomic changes based on the different immunosuppressive strategies. Moreover, the value of specific proteomic signatures on long-term outcomes such as graft survival is limited. Importantly, the analysis did not include patients who received basiliximab, the most commonly used IL-2 receptor antagonist. There were also notable differences in immunosuppression protocols between cohorts, which should be considered when interpreting findings. Furthermore, the one-year time point reflects not only the effect of induction therapy but also the cumulative impact of clinical events throughout the first year. A limitation of our label-free MS approach is the use of a single data-dependent acquisition (DDA) run per sample with a “top-20” strategy. DDA is known to suffer from undersampling, missing data, and variability—particularly when compared to data-independent acquisition (DIA) or targeted techniques—and can limit quantitative precision. We recognize that orthogonal validation (e.g., MRM or PRM workflows with internal standards) would enhance confidence in our findings. Finally, Our study relies exclusively on serum proteomics. While serum proteomics allows minimally invasive longitudinal monitoring and provides valuable systemic insights, it remains limited in proteome depth, dynamic range, and tissue specificity. These constraints should be considered when interpreting our findings. Future studies can leverage technical advances such as data-independent acquisition, single-cell proteomics, top-down and targeted proteomics, and incorporate multi-omics approaches, to better capture tissue-specific and cellular-level signals.

In summary, our findings demonstrate that alemtuzumab is associated with a more profound downregulation of immune and inflammatory proteins and upregulation of proteins like albumin and apoA-I. The downregulation of Fetuin-B in the alemtuzumab group suggests its specific role in the immune changes caused by this depleting agent, compared to the other two immunosuppressive strategies. Interestingly, alemtuzumab and ATG groups exhibited distinct serum proteomic signatures.

## Data Availability

All relevant data is contained within the article: The original contributions presented in the study are included in the article/**Supplementary Material**, further inquiries can be directed to the corresponding author.
