# Supplementary material for: Serum proteomics analysis of lung transplant patients receiving different induction therapies
Source: Front Immunol. 2025 Aug 28;16:1616781. doi: 10.3389/fimmu.2025.1616781 (PMC12422901; doi:10.3389/fimmu.2025.1616781)
Supplement: Supplementary file 1 [file DataSheet1.docx]

**Supplementary Materials**

**Supplementary tables (1-4)**

**Table S1. Maintenance Immunosuppression Protocol.** Overview of immunosuppression regimens used in this study.

| *Table 1 - Maintenance immunosuppression protocol* | Calcineurin Inhibitors | | | | Aprednisolone mg/kg | | Anti-proliferative | |
| --- | --- | --- | --- | --- | --- | --- | --- | --- |
|  | No induction /ATG | | Alemtuzumab | | No induction /ATG | Alemtuzumab | No induction /ATG | Alemtuzumab |
|  | CyA ng/ml | Tacrolimus ng/ml | CyA ng/ml | Tacrolimus ng/ml |  |  | MMF | |
| 0-3 months | 300 - 350 | 15-18 | 200 | 10-12 | 0.3 | 0.2 | 1-1.5g twice a day | - |
| 3-6 months |  | 13-15 |  | 8-10 | 0.2 | 0.15 | 1-1.5g twice a day | - |
| 6-12 months | 250 - 300 | 10-12 | 150 | 6-8 | 0.15 | 0.1 | 1-1.5g twice a day | - |
| 12-24 months | 200 | 8-10 | 150 | 5-7 | 5 mg/d | 5 mg/d | 1-1.5g twice a day | 1-1.5g twice a day |
| >24 months | 100 - 200 | 8 | 100 - 150 | 5 |  |  | 1-1.5g twice a day |  |

**Table S2. Differentially Expressed Serum Proteins: alemtuzumab vs. No-Induction Therapy Pre-Transplantation**. The table lists significantly differentially expressed serum proteins in the comparison of alemtuzumab and no-induction therapy at the pre-transplantation stage. Proteins are classified into upregulated and downregulated, with gene symbols, full protein descriptions, and their respective log2 fold changes (log2FC), p-values, and false discovery rates (FDR) for each protein presented.

| **Alemtuzumab T1 vs. No-induction T1** | | | | |
| --- | --- | --- | --- | --- |
| **Upregulated Proteins** | | | | |
| **Gene** | **Description** | **p-value** | **FDR** | **log2FC** |
| IGLC3 | Immunoglobulin lambda constant 3 OS=Homo sapiens OX=9606 GN=IGLC3 PE=1 SV=1 | 4.61E-07 | 3.36E-05 | 2,60E+00 |
| IGHA1 | Immunoglobulin heavy constant alpha 1 OS=Homo sapiens OX=9606 GN=IGHA1 PE=1 SV=2 | 0.00014 | 0.00232 | 1,26E+00 |
| IGFBP2 | Insulin-like growth factor-binding protein 2 OS=Homo sapiens OX=9606 GN=IGFBP2 PE=1 SV=2 | 0.00015 | 0.00239 | 1,21E+00 |
| JCHAIN | Immunoglobulin J chain OS=Homo sapiens OX=9606 GN=JCHAIN PE=1 SV=4 | 0.00027 | 0.00358 | 1,72E+00 |
| IGHM | Immunoglobulin heavy constant mu OS=Homo sapiens OX=9606 GN=IGHM PE=1 SV=4 | 0.00033 | 0.00419 | 1,41E+00 |
| LTBP1 | Latent-transforming growth factor beta-binding protein 1 OS=Homo sapiens OX=9606 GN=LTBP1 PE=1 SV=4 | 0.00039 | 0.00443 | 1,23E+00 |
| IGFBP4 | Insulin-like growth factor-binding protein 4 OS=Homo sapiens OX=9606 GN=IGFBP4 PE=1 SV=2 | 0.00073 | 0.00669 | 9,23E-01 |
| CAVIN2 | Caveolae-associated protein 2 OS=Homo sapiens OX=9606 GN=CAVIN2 PE=1 SV=3 | 0.00129 | 0.01024 | 2,29E+00 |
| IGHG2 | Immunoglobulin heavy constant gamma 2 OS=Homo sapiens OX=9606 GN=IGHG2 PE=1 SV=2 | 0.00154 | 0.01146 | 1,27E+00 |
| IGLV1-47 | Immunoglobulin lambda variable 1-47 OS=Homo sapiens OX=9606 GN=IGLV1-47 PE=1 SV=2 | 0.0021 | 0.01415 | 1,76E+00 |
| ALB | Albumin OS=Homo sapiens OX=9606 GN=ALB PE=1 SV=2 | 0.00259 | 0.01583 | 9,79E-01 |
| KNG1 | Isoform LMW of Kininogen-1 OS=Homo sapiens OX=9606 GN=KNG1 | 0.00309 | 0.01841 | 8,11E-01 |
| SBSN | Suprabasin OS=Homo sapiens OX=9606 GN=SBSN PE=1 SV=2 | 0.00404 | 0.02204 | 1,51E+00 |
| IGKC | Immunoglobulin kappa constant OS=Homo sapiens OX=9606 GN=IGKC PE=1 SV=2 | 0.00469 | 0.02402 | 8,95E-01 |
| PRG4 | Isoform C of Proteoglycan 4 OS=Homo sapiens OX=9606 GN=PRG4 | 0.00495 | 0.0247 | 1,23E+00 |
| ORM1 | Alpha-1-acid glycoprotein 1 OS=Homo sapiens OX=9606 GN=ORM1 PE=1 SV=2 | 0.00515 | 0.02518 | 1,20E+00 |
| ICAM2 | Intercellular adhesion molecule 2 OS=Homo sapiens OX=9606 GN=ICAM2 PE=1 SV=2 | 0.00519 | 0.02518 | 1,21E+00 |
| IGLL5 | Immunoglobulin lambda-like polypeptide 5 OS=Homo sapiens OX=9606 GN=IGLL5 PE=2 SV=2 | 0.00562 | 0.0269 | 1,35E+00 |
| SLPI | Antileukoproteinase OS=Homo sapiens OX=9606 GN=SLPI PE=1 SV=2 | 0.00667 | 0.02962 | 2,34E+00 |
| IGHG3 | Immunoglobulin heavy constant gamma 3 OS=Homo sapiens OX=9606 GN=IGHG3 PE=1 SV=2 | 0.00769 | 0.03331 | 9,56E-01 |
| MENT | Protein MENT OS=Homo sapiens OX=9606 GN=MENT PE=1 SV=1 | 0.00858 | 0.03673 | 9,86E-01 |
| ICAM1 | Intercellular adhesion molecule 1 OS=Homo sapiens OX=9606 GN=ICAM1 PE=1 SV=2 | 0.00966 | 0.03996 | 8,21E-01 |
| SSC5D | Soluble scavenger receptor cysteine-rich domain-containing protein SSC5D OS=Homo sapiens OX=9606 GN=SSC5D PE=1 SV=3 | 0.01008 | 0.04078 | 1,53E+00 |
| HP | Haptoglobin OS=Homo sapiens OX=9606 GN=HP PE=1 SV=1 | 0.01128 | 0.04413 | 1,19E+00 |
| **Downregulated Proteins** | | | | |
| SERPING1 | Plasma protease C1 inhibitor OS=Homo sapiens OX=9606 GN=SERPING1 PE=1 SV=2 | 1.58E-08 | 5.77E-06 | -1.76E+00 |
| APOB | Apolipoprotein B-100 OS=Homo sapiens OX=9606 GN=APOB PE=1 SV=2 | 2.80E-07 | 3.36E-05 | -1.37E+00 |
| ITIH2 | Inter-alpha-trypsin inhibitor heavy chain H2 OS=Homo sapiens OX=9606 GN=ITIH2 PE=1 SV=2 | 3.99E-07 | 3.36E-05 | -9.26E-01 |
| PON1 | Serum paraoxonase/arylesterase 1 OS=Homo sapiens OX=9606 GN=PON1 PE=1 SV=3 | 4.10E-07 | 3.36E-05 | -1.53E+00 |
| ITIH1 | Inter-alpha-trypsin inhibitor heavy chain H1 OS=Homo sapiens OX=9606 GN=ITIH1 PE=1 SV=3 | 1.77E-06 | 0.00011 | -1.30E+00 |
| GPLD1 | Phosphatidylinositol-glycan-specific phospholipase D OS=Homo sapiens OX=9606 GN=GPLD1 PE=1 SV=3 | 4.58E-06 | 0.00024 | -1.22E+00 |
| AFM | Afamin OS=Homo sapiens OX=9606 GN=AFM PE=1 SV=1 | 8.19E-06 | 0.00037 | -7.95E-01 |
| APOE | Apolipoprotein E OS=Homo sapiens OX=9606 GN=APOE PE=1 SV=1 | 9.31E-06 | 0.00038 | -9.20E-01 |
| CP | Ceruloplasmin OS=Homo sapiens OX=9606 GN=CP PE=1 SV=1 | 1.09E-05 | 0.00038 | -1.13E+00 |
| IGFALS | Insulin-like growth factor-binding protein complex acid labile subunit OS=Homo sapiens OX=9606 GN=IGFALS PE=1 SV=1 | 1.14E-05 | 0.00038 | -1.41E+00 |
| C4A | Complement C4-A OS=Homo sapiens OX=9606 GN=C4A PE=1 SV=2 | 1.34E-05 | 0.00041 | -2.17E+00 |
| SERPINF2 | Alpha-2-antiplasmin OS=Homo sapiens OX=9606 GN=SERPINF2 PE=1 SV=3 | 1.48E-05 | 0.00041 | -9.19E-01 |
| SERPIND1 | Heparin cofactor 2 OS=Homo sapiens OX=9606 GN=SERPIND1 PE=1 SV=3 | 1.75E-05 | 0.00045 | -1.14E+00 |
| C3 | Complement C3 OS=Homo sapiens OX=9606 GN=C3 PE=1 SV=2 | 2.01E-05 | 0.00046 | -7.88E-01 |
| HPX | Hemopexin OS=Homo sapiens OX=9606 GN=HPX PE=1 SV=2 | 1.99E-05 | 0.00046 | -9.31E-01 |
| ITIH3 | Inter-alpha-trypsin inhibitor heavy chain H3 OS=Homo sapiens OX=9606 GN=ITIH3 PE=1 SV=2 | 2.93E-05 | 0.00063 | -9.37E-01 |
| SERPINA6 | Corticosteroid-binding globulin OS=Homo sapiens OX=9606 GN=SERPINA6 PE=1 SV=1 | 3.81E-05 | 0.00077 | -1,44E+00 |
| CPB2 | Carboxypeptidase B2 OS=Homo sapiens OX=9606 GN=CPB2 PE=1 SV=2 | 5.22E-05 | 0.001 | -9.70E-01 |
| C4B_2 | Complement C4-B OS=Homo sapiens OX=9606 GN=C4B_2 PE=1 SV=2 | 5.83E-05 | 0.00106 | -1.50E+00 |
| FBLN1 | Fibulin-1 OS=Homo sapiens OX=9606 GN=FBLN1 PE=1 SV=4 | 8.28E-05 | 0.00143 | -2.12E+00 |
| FN1 | Isoform 1 of Fibronectin OS=Homo sapiens OX=9606 GN=FN1 | 0.00016 | 0.00249 | -1.22E+00 |
| LYVE1 | Lymphatic vessel endothelial hyaluronic acid receptor 1 OS=Homo sapiens OX=9606 GN=LYVE1 PE=1 SV=2 | 0.00017 | 0.00252 | -1.30E+00 |
| CLEC3B | Tetranectin OS=Homo sapiens OX=9606 GN=CLEC3B PE=1 SV=3 | 0.00026 | 0.00358 | -1.20E+00 |
| SERPINC1 | Antithrombin-III OS=Homo sapiens OX=9606 GN=SERPINC1 PE=1 SV=1 | 0.00031 | 0.00407 | -6.27E-01 |
| TTR | Transthyretin OS=Homo sapiens OX=9606 GN=TTR PE=1 SV=1 | 0.00035 | 0.00428 | -1.29E+00 |
| C5 | Complement C5 OS=Homo sapiens OX=9606 GN=C5 PE=1 SV=4 | 0.00039 | 0.00443 | -9.62E-01 |
| C1QA | Complement C1q subcomponent subunit A OS=Homo sapiens OX=9606 GN=C1QA PE=1 SV=2 | 0.00047 | 0.00505 | -1.16E+00 |
| SERPINA4 | Kallistatin OS=Homo sapiens OX=9606 GN=SERPINA4 PE=1 SV=3 | 0.00047 | 0.00505 | -1.09E+00 |
| LCAT | Phosphatidylcholine-sterol acyltransferase OS=Homo sapiens OX=9606 GN=LCAT PE=1 SV=1 | 0.00049 | 0.00509 | -1.20E+00 |
| CFH | Complement factor H OS=Homo sapiens OX=9606 GN=CFH PE=1 SV=4 | 0.0005 | 0.00509 | -7.03E-01 |
| CLU | Isoform 6 of Clusterin OS=Homo sapiens OX=9606 GN=CLU | 0.00057 | 0.00556 | -6.73E-01 |
| C1RL | Complement C1r subcomponent-like protein OS=Homo sapiens OX=9606 GN=C1RL PE=1 SV=2 | 0.00058 | 0.00556 | -9.00E-01 |
| AGT | Angiotensinogen OS=Homo sapiens OX=9606 GN=AGT PE=1 SV=3 | 0.00067 | 0.00628 | -1.03E+00 |
| ENPP2 | Ectonucleotide pyrophosphatase/phosphodiesterase family member 2 OS=Homo sapiens OX=9606 GN=ENPP2 PE=1 SV=3 | 0.00076 | 0.00678 | -2.00E+00 |
| APOA4 | Apolipoprotein A-IV OS=Homo sapiens OX=9606 GN=APOA4 PE=1 SV=4 | 0.00109 | 0.00948 | -7.48E-01 |
| C8G | Complement component C8 gamma chain OS=Homo sapiens OX=9606 GN=C8G PE=1 SV=3 | 0.00118 | 0.00977 | -6.87E-01 |
| SERPINF1 | Pigment epithelium-derived factor OS=Homo sapiens OX=9606 GN=SERPINF1 PE=1 SV=4 | 0.00118 | 0.00977 | -9.20E-01 |
| C4BPA | C4b-binding protein alpha chain OS=Homo sapiens OX=9606 GN=C4BPA PE=1 SV=2 | 0.00126 | 0.01018 | -7.51E-01 |
| LTF | Lactotransferrin OS=Homo sapiens OX=9606 GN=LTF PE=1 SV=6 | 0.0014 | 0.01085 | -1.29E+00 |
| CNDP1 | Beta-Ala-His dipeptidase OS=Homo sapiens OX=9606 GN=CNDP1 PE=1 SV=5 | 0.00152 | 0.01146 | -1.21E+00 |
| SERPINA3 | Alpha-1-antichymotrypsin OS=Homo sapiens OX=9606 GN=SERPINA3 PE=1 SV=2 | 0.00162 | 0.01154 | -9.25E-01 |
| ALDOA | Fructose-bisphosphate aldolase A OS=Homo sapiens OX=9606 GN=ALDOA PE=1 SV=2 | 0.00161 | 0.01154 | -1.10E+00 |
| SERPINA5 | Plasma serine protease inhibitor OS=Homo sapiens OX=9606 GN=SERPINA5 PE=1 SV=3 | 0.002 | 0.01399 | -1.22E+00 |
| BTD | Biotinidase OS=Homo sapiens OX=9606 GN=BTD PE=1 SV=2 | 0.00214 | 0.01415 | -1.03E+00 |
| LYZ | Lysozyme C OS=Homo sapiens OX=9606 GN=LYZ PE=1 SV=1 | 0.00212 | 0.01415 | -1.13E+00 |
| PROS1 | Vitamin K-dependent protein S OS=Homo sapiens OX=9606 GN=PROS1 PE=1 SV=1 | 0.00226 | 0.01461 | -4.82E-01 |
| FCGBP | IgGFc-binding protein OS=Homo sapiens OX=9606 GN=FCGBP PE=1 SV=3 | 0.00229 | 0.01461 | -1.06E+00 |
| SERPINA7 | Thyroxine-binding globulin OS=Homo sapiens OX=9606 GN=SERPINA7 PE=1 SV=2 | 0.00233 | 0.01463 | -1.18E+00 |
| F13B | Coagulation factor XIII B chain OS=Homo sapiens OX=9606 GN=F13B PE=1 SV=3 | 0.00261 | 0.01583 | -1.31E+00 |
| C1QB | Complement C1q subcomponent subunit B OS=Homo sapiens OX=9606 GN=C1QB PE=1 SV=3 | 0.00315 | 0.01848 | -7.89E-01 |
| APOA1 | Apolipoprotein A-I OS=Homo sapiens OX=9606 GN=APOA1 PE=1 SV=1 | 0.00351 | 0.01994 | -1.00E+00 |
| F11 | Coagulation factor XI OS=Homo sapiens OX=9606 GN=F11 PE=1 SV=1 | 0.00349 | 0.01994 | -6.90E-01 |
| CRTAC1 | Cartilage acidic protein 1 OS=Homo sapiens OX=9606 GN=CRTAC1 PE=1 SV=2 | 0.00381 | 0.02136 | -1.02E+00 |
| BLVRB | Flavin reductase (NADPH) OS=Homo sapiens OX=9606 GN=BLVRB PE=1 SV=3 | 0.00406 | 0.02204 | -1.39E+00 |
| CFD | Complement factor D OS=Homo sapiens OX=9606 GN=CFD PE=1 SV=5 | 0.00438 | 0.02342 | -6.85E-01 |
| NID1 | Nidogen-1 OS=Homo sapiens OX=9606 GN=NID1 PE=1 SV=3 | 0.00459 | 0.02402 | -1.10E+00 |
| SERPINA10 | Protein Z-dependent protease inhibitor OS=Homo sapiens OX=9606 GN=SERPINA10 PE=1 SV=1 | 0.00464 | 0.02402 | -8.68E-01 |
| LCP1 | Plastin-2 OS=Homo sapiens OX=9606 GN=LCP1 PE=1 SV=6 | 0.0049 | 0.0247 | -1.16E+00 |
| PTGDS | Prostaglandin-H2 D-isomerase OS=Homo sapiens OX=9606 GN=PTGDS PE=1 SV=1 | 0.00583 | 0.02755 | -1.14E+00 |
| PRSS1 | Serine protease 1 OS=Homo sapiens OX=9606 GN=PRSS1 PE=1 SV=1 | 0.00597 | 0.02786 | -1.72E+00 |
| MMRN1 | Multimerin-1 OS=Homo sapiens OX=9606 GN=MMRN1 PE=1 SV=3 | 0.00611 | 0.02814 | -9.47E-01 |
| MMP2 | 72 kDa type IV collagenase OS=Homo sapiens OX=9606 GN=MMP2 PE=1 SV=2 | 0.00645 | 0.02935 | -1.25E+00 |
| CPN2 | Carboxypeptidase N subunit 2 OS=Homo sapiens OX=9606 GN=CPN2 PE=1 SV=3 | 0.00655 | 0.02942 | -5.50E-01 |
| PROC | Vitamin K-dependent protein C OS=Homo sapiens OX=9606 GN=PROC PE=1 SV=1 | 0.00702 | 0.03079 | -6.28E-01 |
| MASP2 | Mannan-binding lectin serine protease 2 OS=Homo sapiens OX=9606 GN=MASP2 PE=1 SV=4 | 0.00922 | 0.03901 | -7.56E-01 |
| NCAM1 | Isoform 2 of Neural cell adhesion molecule 1 OS=Homo sapiens OX=9606 GN=NCAM1 | 0.00958 | 0.03996 | -8.87E-01 |
| NRP1 | Neuropilin-1 OS=Homo sapiens OX=9606 GN=NRP1 PE=1 SV=3 | 0.00992 | 0.04056 | -1.10E+00 |
| C1R | Complement C1r subcomponent OS=Homo sapiens OX=9606 GN=C1R PE=1 SV=2 | 0.01092 | 0.04369 | -5.43E-01 |
| APOD | Apolipoprotein D OS=Homo sapiens OX=9606 GN=APOD PE=1 SV=1 | 0.01115 | 0.0441 | -5.92E-01 |
| PEBP4 | Phosphatidylethanolamine-binding protein 4 OS=Homo sapiens OX=9606 GN=PEBP4 PE=1 SV=3 | 0.01151 | 0.04455 | -1.58E+00 |

**Table S3. Differentially Expressed Serum Proteins in alemtuzumab vs. ATG Therapies Pre-Transplantation**. The table provides a list of serum proteins with significant differential expression between alemtuzumab and ATG therapies at the pre-transplantation stage. Proteins are categorized as upregulated or downregulated, with their corresponding gene symbols, detailed protein descriptions, log2 fold changes (log2FC), p-values, and false discovery rates (FDR) provided for each protein.

| **Alemtuzumab T1 vs. ATG T1** | | | | |
| --- | --- | --- | --- | --- |
| **Upregulated Proteins** | | | | |
| **Gene** | **Description** | **p-value** | **FDR** | **log2FC** |
| IGLC3 | Immunoglobulin lambda constant 3 OS=Homo sapiens OX=9606 GN=IGLC3 PE=1 SV=1 | 3.71E-05 | 0.00337 | 2.6132308 |
| JCHAIN | Immunoglobulin J chain OS=Homo sapiens OX=9606 GN=JCHAIN PE=1 SV=4 | 9.68E-05 | 0.00538 | 2.27864407 |
| IGLL5 | Immunoglobulin lambda-like polypeptide 5 OS=Homo sapiens OX=9606 GN=IGLL5 PE=2 SV=2 | 0.00049 | 0.01111 | 2.03866025 |
| GLIPR2 | Golgi-associated plant pathogenesis-related protein 1 OS=Homo sapiens OX=9606 GN=GLIPR2 PE=1 SV=3 | 0.00102 | 0.01479 | 2.21897689 |
| PDLIM1 | PDZ and LIM domain protein 1 OS=Homo sapiens OX=9606 GN=PDLIM1 PE=1 SV=4 | 0.00134 | 0.01841 | 2.14746588 |
| IGHA1 | Immunoglobulin heavy constant alpha 1 OS=Homo sapiens OX=9606 GN=IGHA1 PE=1 SV=2 | 0.00262 | 0.02645 | 1.19679125 |
| IGHM | Immunoglobulin heavy constant mu OS=Homo sapiens OX=9606 GN=IGHM PE=1 SV=4 | 0.00296 | 0.0276 | 1.40138799 |
| **Downregulated Proteins** | | | | |
| ALDOA | Fructose-bisphosphate aldolase A OS=Homo sapiens OX=9606 GN=ALDOA PE=1 SV=2 | 4.08E-06 | 0.00074 | -1.8928496 |
| GPLD1 | Phosphatidylinositol-glycan-specific phospholipase D OS=Homo sapiens OX=9606 GN=GPLD1 PE=1 SV=3 | 2.88E-06 | 0.00074 | -1.5102812 |
| SERPING1 | Plasma protease C1 inhibitor OS=Homo sapiens OX=9606 GN=SERPING1 PE=1 SV=2 | 3.33E-05 | 0.00337 | -1.5267252 |
| CP | Ceruloplasmin OS=Homo sapiens OX=9606 GN=CP PE=1 SV=1 | 9.16E-05 | 0.00538 | -1.2047067 |
| HPX | Hemopexin OS=Homo sapiens OX=9606 GN=HPX PE=1 SV=2 | 0.0001 | 0.00538 | -1.0188455 |
| APOB | Apolipoprotein B-100 OS=Homo sapiens OX=9606 GN=APOB PE=1 SV=2 | 0.00012 | 0.00538 | -1.216802 |
| C4A | Complement C4-A OS=Homo sapiens OX=9606 GN=C4A PE=1 SV=2 | 0.00013 | 0.00538 | -2.2647004 |
| ITIH1 | Inter-alpha-trypsin inhibitor heavy chain H1 OS=Homo sapiens OX=9606 GN=ITIH1 PE=1 SV=3 | 0.00024 | 0.00857 | -1.1897805 |
| C8G | Complement component C8 gamma chain OS=Homo sapiens OX=9606 GN=C8G PE=1 SV=3 | 0.00031 | 0.01036 | -0.9252955 |
| TTR | Transthyretin OS=Homo sapiens OX=9606 GN=TTR PE=1 SV=1 | 0.0004 | 001058 | -1.5445876 |
| ITIH2 | Inter-alpha-trypsin inhibitor heavy chain H2 OS=Homo sapiens OX=9606 GN=ITIH2 PE=1 SV=2 | 0.00036 | 0.01058 | -0.7736452 |
| FBLN1 | Fibulin-1 OS=Homo sapiens OX=9606 GN=FBLN1 PE=1 SV=4 | 0.00042 | 0.01058 | -2.2879979 |
| LYZ | Lysozyme C OS=Homo sapiens OX=9606 GN=LYZ PE=1 SV=1 | 0.00044 | 0.01058 | -1.5741652 |
| CFH | Complement factor H OS=Homo sapiens OX=9606 GN=CFH PE=1 SV=4 | 0.0006 | 0.01193 | -0.8388944 |
| IGFALS | Insulin-like growth factor-binding protein complex acid labile subunit OS=Homo sapiens OX=9606 GN=IGFALS PE=1 SV=1 | 0.00059 | 0.01193 | -1.3184422 |
| LYVE1 | Lymphatic vessel endothelial hyaluronic acid receptor 1 OS=Homo sapiens OX=9606 GN=LYVE1 PE=1 SV=2 | 0.00062 | 0.01193 | -1.4240536 |
| C3 | Complement C3 OS=Homo sapiens OX=9606 GN=C3 PE=1 SV=2 | 0.00086 | 0.01374 | -0.7370883 |
| FN1 | Isoform 1 of Fibronectin OS=Homo sapiens OX=9606 GN=FN1 | 0.0008 | 0.01374 | -1.306768 |
| SERPINF2 | Alpha-2-antiplasmin OS=Homo sapiens OX=9606 GN=SERPINF2 PE=1 SV=3 | 0.00087 | 0.01374 | -0.8442001 |
| ECM1 | Extracellular matrix protein 1 OS=Homo sapiens OX=9606 GN=ECM1 PE=1 SV=2 | 0.00085 | 0.01374 | -1.0509552 |
| SERPINA5 | Plasma serine protease inhibitor OS=Homo sapiens OX=9606 GN=SERPINA5 PE=1 SV=3 | 0.00092 | 0.01393 | -1.5659398 |
| SERPINA6 | Corticosteroid-binding globulin OS=Homo sapiens OX=9606 GN=SERPINA6 PE=1 SV=1 | 0.0014 | 0.01841 | -1.3344157 |
| CFHR2 | Complement factor H-related protein 2 OS=Homo sapiens OX=9606 GN=CFHR2 PE=1 SV=1 | 0.00142 | 0.01841 | -1.2145586 |
| C1QA | Complement C1q subcomponent subunit A OS=Homo sapiens OX=9606 GN=C1QA PE=1 SV=2 | 0.00159 | 0.01925 | -1.2606695 |
| ITIH3 | Inter-alpha-trypsin inhibitor heavy chain H3 OS=Homo sapiens OX=9606 GN=ITIH3 PE=1 SV=2 | 0.00156 | 0.01925 | -0.8485454 |
| VTN | Vitronectin OS=Homo sapiens OX=9606 GN=VTN PE=1 SV=1 | 0.00174 | 0.02046 | -0.5118367 |
| AGT | Angiotensinogen OS=Homo sapiens OX=9606 GN=AGT PE=1 SV=3 | 0.00188 | 0.02139 | -1.1303926 |
| APCS | Serum amyloid P-component OS=Homo sapiens OX=9606 GN=APCS PE=1 SV=2 | 0.00208 | 0.02279 | -1.9349482 |
| SERPIND1 | Heparin cofactor 2 OS=Homo sapiens OX=9606 GN=SERPIND1 PE=1 SV=3 | 0.00213 | 0.02279 | -0.9674868 |
| C4B_2 | Complement C4-B OS=Homo sapiens OX=9606 GN=C4B_2 PE=1 SV=2 | 0.00221 | 0.02297 | -1.3668466 |
| C1QB | Complement C1q subcomponent subunit B OS=Homo sapiens OX=9606 GN=C1QB PE=1 SV=3 | 0.00286 | 0.02743 | -0.9622856 |
| LCAT | Phosphatidylcholine-sterol acyltransferase OS=Homo sapiens OX=9606 GN=LCAT PE=1 SV=1 | 0.00282 | 0.02743 | -1.2329866 |
| C5 | Complement C5 OS=Homo sapiens OX=9606 GN=C5 PE=1 SV=4 | 0.00331 | 0.03008 | -0.957277 |
| MAN1A1 | Mannosyl-oligosaccharide 1,2-alpha-mannosidase IA OS=Homo sapiens OX=9606 GN=MAN1A1 PE=1 SV=3 | 0.00339 | 0.03008 | -1.3764117 |
| AFM | Afamin OS=Homo sapiens OX=9606 GN=AFM PE=1 SV=1 | 0.00354 | 0.03071 | -0.6176482 |
| ENPP2 | Ectonucleotide pyrophosphatase/phosphodiesterase family member 2 OS=Homo sapiens OX=9606 GN=ENPP2 PE=1 SV=3 | 0.00459 | 0.03889 | -2.0830015 |
| TFRC | Transferrin receptor protein 1 OS=Homo sapiens OX=9606 GN=TFRC PE=1 SV=2 | 0.00534 | 0.04414 | -1.3438866 |
| SERPINA7 | Thyroxine-binding globulin OS=Homo sapiens OX=9606 GN=SERPINA7 PE=1 SV=2 | 0.00554 | 0.04482 | -1.2913508 |
| C1RL | Complement C1r subcomponent-like protein OS=Homo sapiens OX=9606 GN=C1RL PE=1 SV=2 | 0.00571 | 0.04517 | -0.86777 |
| C1R | Complement C1r subcomponent OS=Homo sapiens OX=9606 GN=C1R PE=1 SV=2 | 0.00631 | 0.04782 | -0.7061378 |
| LTF | Lactotransferrin OS=Homo sapiens OX=9606 GN=LTF PE=1 SV=6 | 0.00628 | 0.04782 | -1.3241822 |
| AZGP1 | Zinc-alpha-2-glycoprotein OS=Homo sapiens OX=9606 GN=AZGP1 PE=1 SV=2 | 0.00644 | 0.04782 | -0.9209775 |

| **Group/Time point** | **Min.** | **Quartile1** | **Median** | **Mean** | **Quartile3** | **Maximum** | **SD** | **Total Values** |
| --- | --- | --- | --- | --- | --- | --- | --- | --- |
| No induction | 264 | 309 | 331 | 326 | 345 | 380 | 26 | 61 |
| ATG | 172 | 290 | 310 | 311 | 338 | 377 | 41 | 32 |
| Alemtuzumab | 93 | 297 | 310 | 313 | 336 | 378 | 36 | 76 |
| No induction-T1 | 276 | 312 | 329 | 327 | 344 | 380 | 24 | 32 |
| ATG-T1 | 285 | 306 | 332 | 327 | 339 | 367 | 22 | 18 |
| Alemtuzumab-T1 | 93 | 299 | 314 | 317 | 339 | 373 | 42 | 47 |
| No induction-T2 | 264 | 298 | 331 | 324 | 348 | 374 | 29 | 29 |
| ATG-T2 | 172 | 274 | 289 | 291 | 310 | 377 | 51 | 14 |
| Alemtuzumab-T2 | 269 | 293 | 302 | 307 | 313 | 378 | 24 | 29 |

**Table S4. Distribution of identified proteins per group and time point.** The table summarizes the number of proteins identified across three treatment groups (No induction, ATG, alemtuzumab) and two time points (T1 and T2) for each group. The statistical distribution for each group and time point is presented, including the minimum, first quartile (Q1), median, mean, third quartile (Q3), maximum, and standard deviation (SD). The total number of values analyzed for each condition is also provided.

**Supplementary Figures (S1-S9)**

**
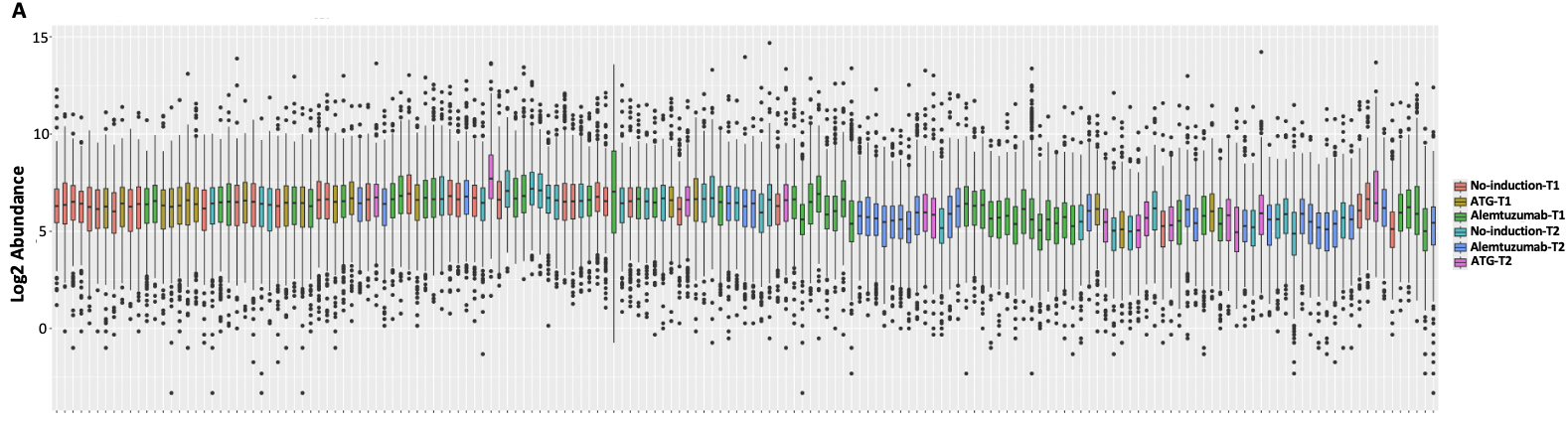
**

**
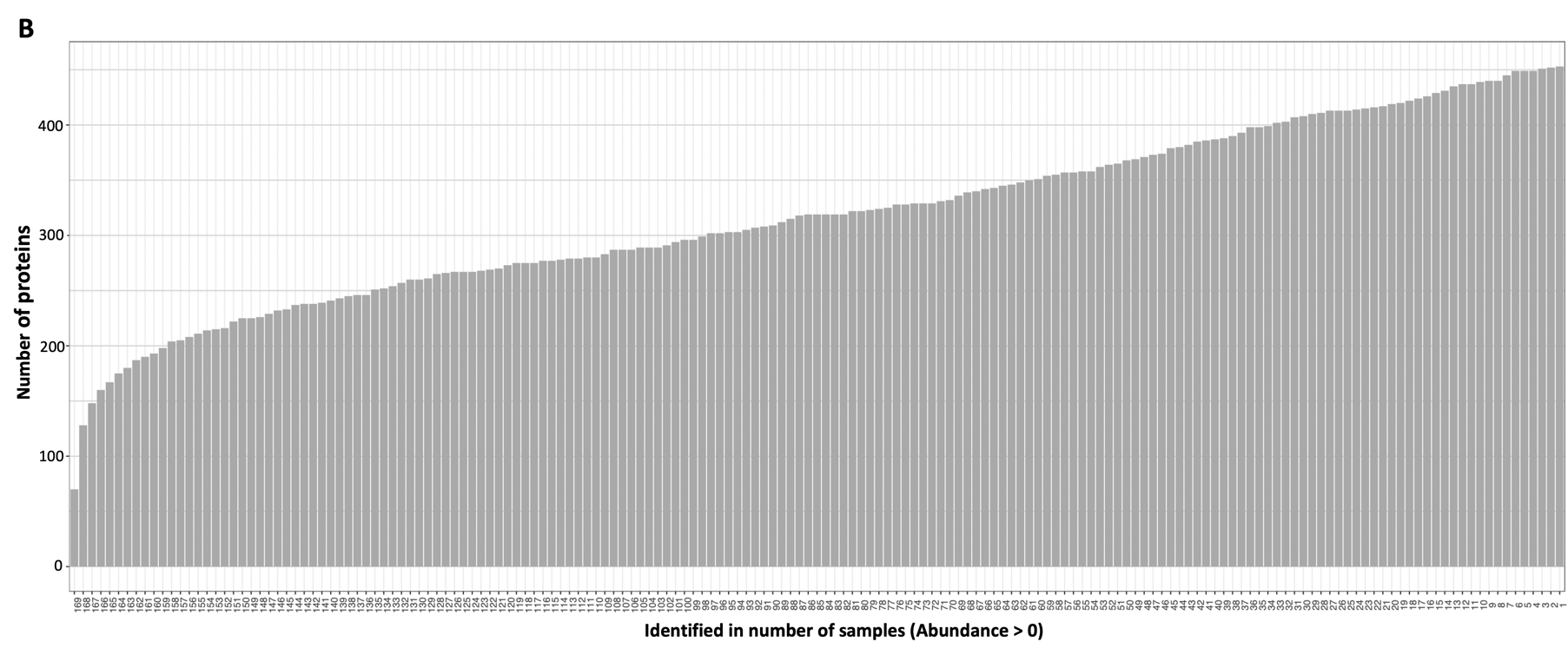
**

**
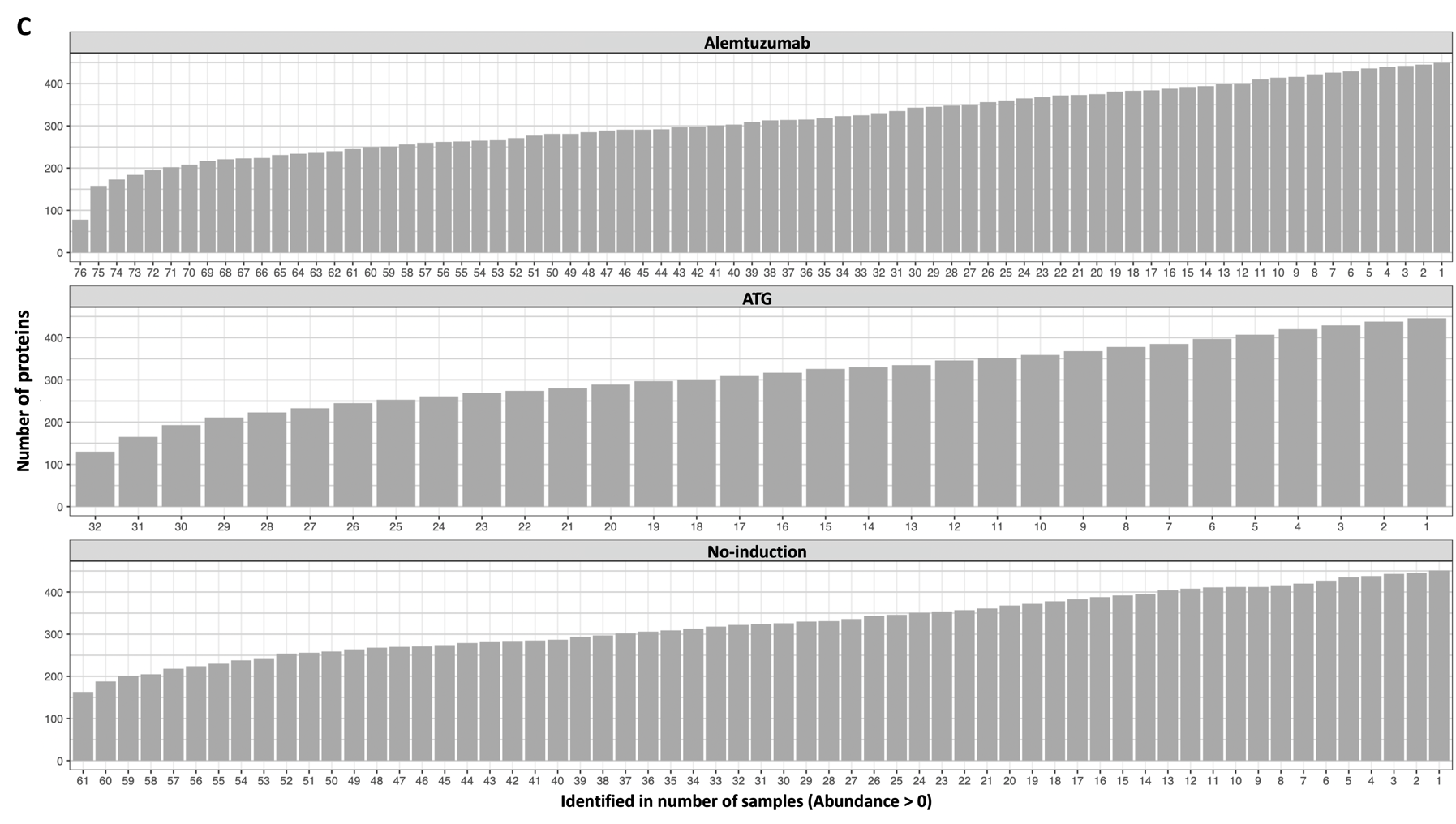
**

**
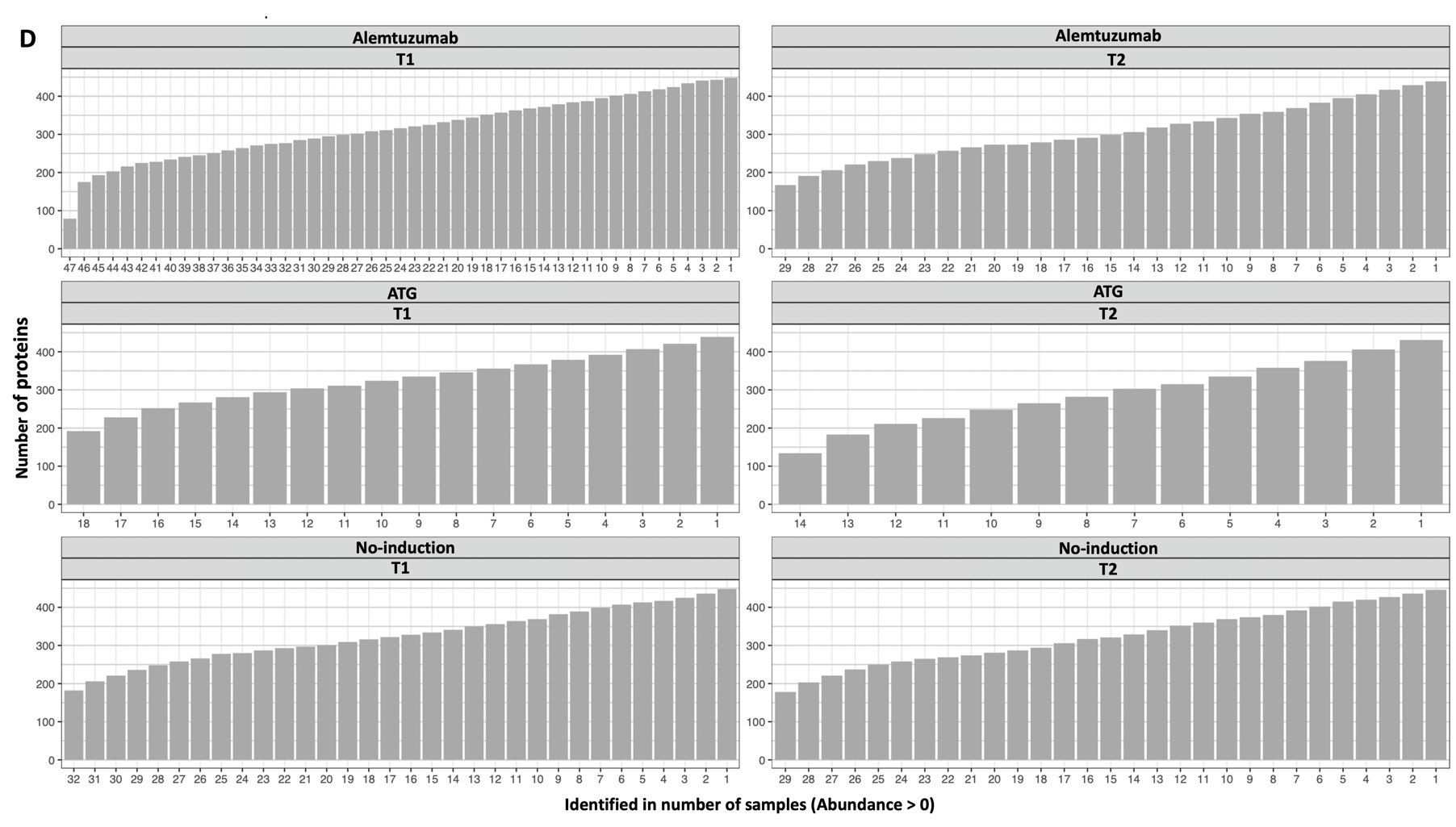
**

**Figure S1. Protein abundance and identification statistics across treatment groups and time points.**
**A)** Box plot of Log2-transformed protein abundances across all samples in each group (No-induction, ATG, alemtuzumab) at T1 (pre-transplant) and T2 (12 months post-transplant). Box centers indicate medians; whiskers span the 10–90th percentile; individual points represent outliers. This visualization helps assess overall data consistency and comparability between groups. **B)** Histogram of protein detection frequency across all samples. The x‑axis shows how many samples a protein was detected in (abundance > 0), and the y‑axis shows the number of proteins found in that many samples. This plot illustrates proteome coverage and sample-to-sample consistency. **C)** Group-level detection histograms. Three panels (alemtuzumab, ATG, No-induction) show the distribution of the number of proteins detected in each treatment group, regardless of time point. The x‑axis indicates how many samples within each group contain a given protein; the y‑axis reflects the count of such proteins. **D)** Group-and-time-point detection histograms, separated by both treatment and time point. This highlights changes in protein detection consistency within each subgroup over the course of the study.

**
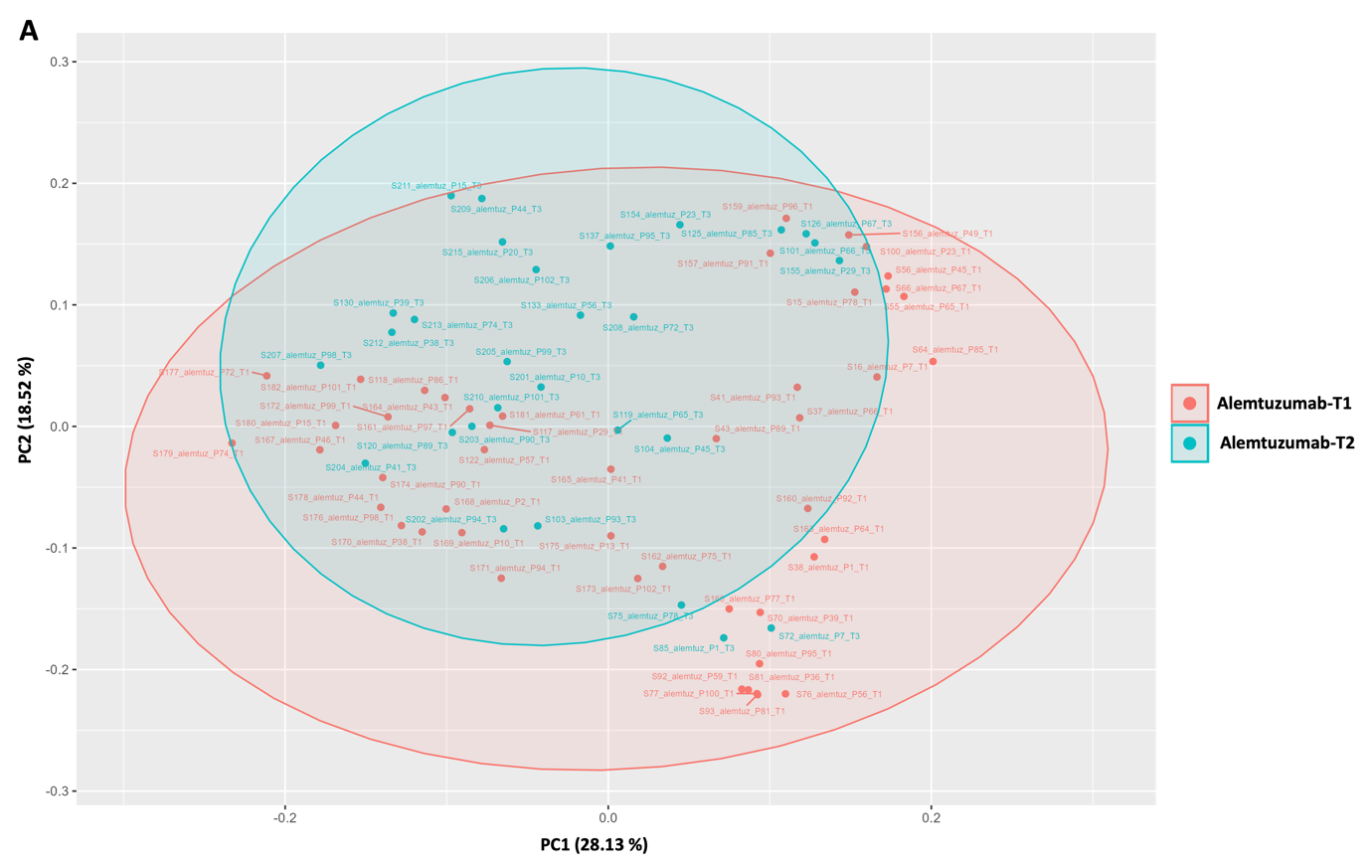
**

**
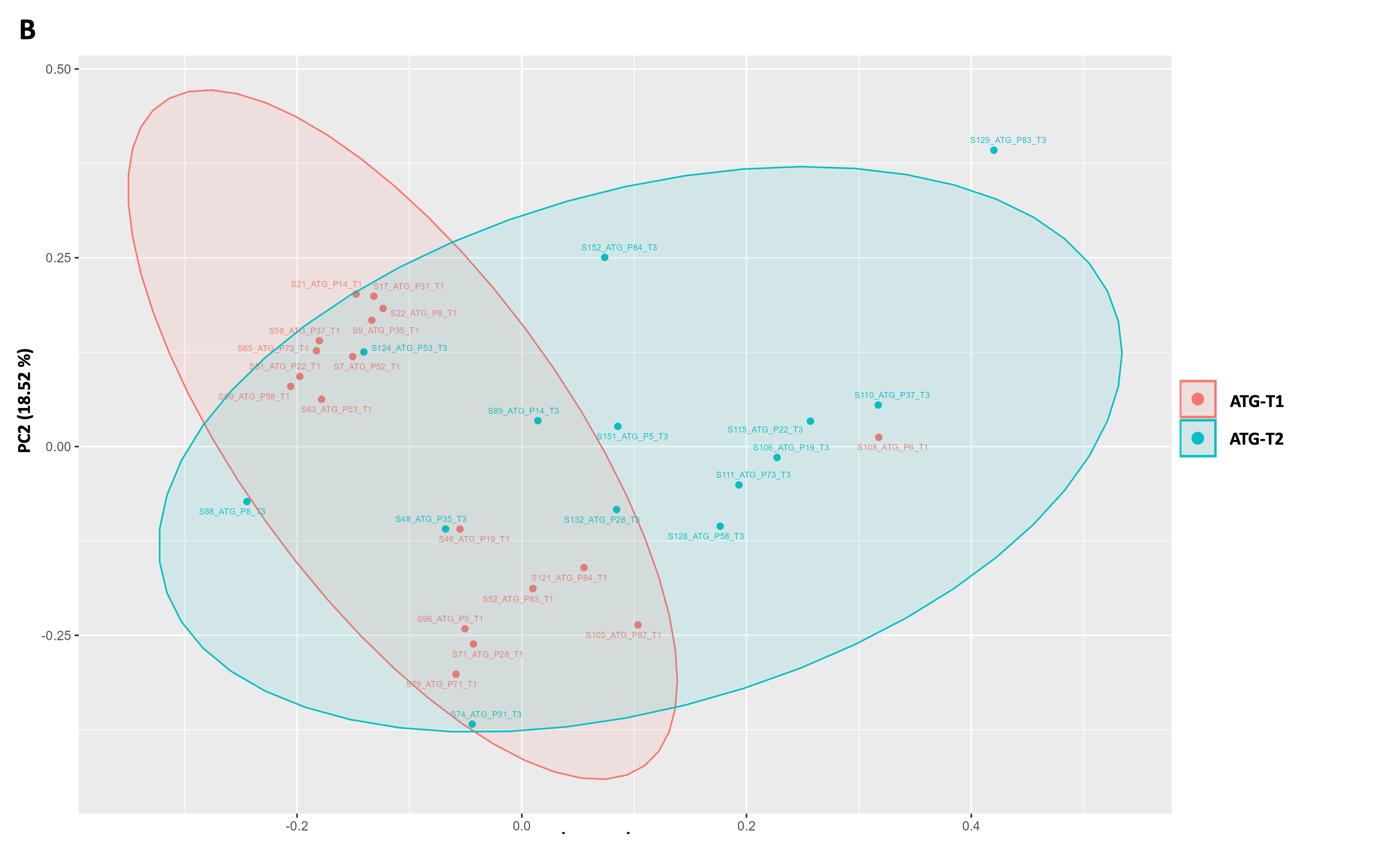
**

**
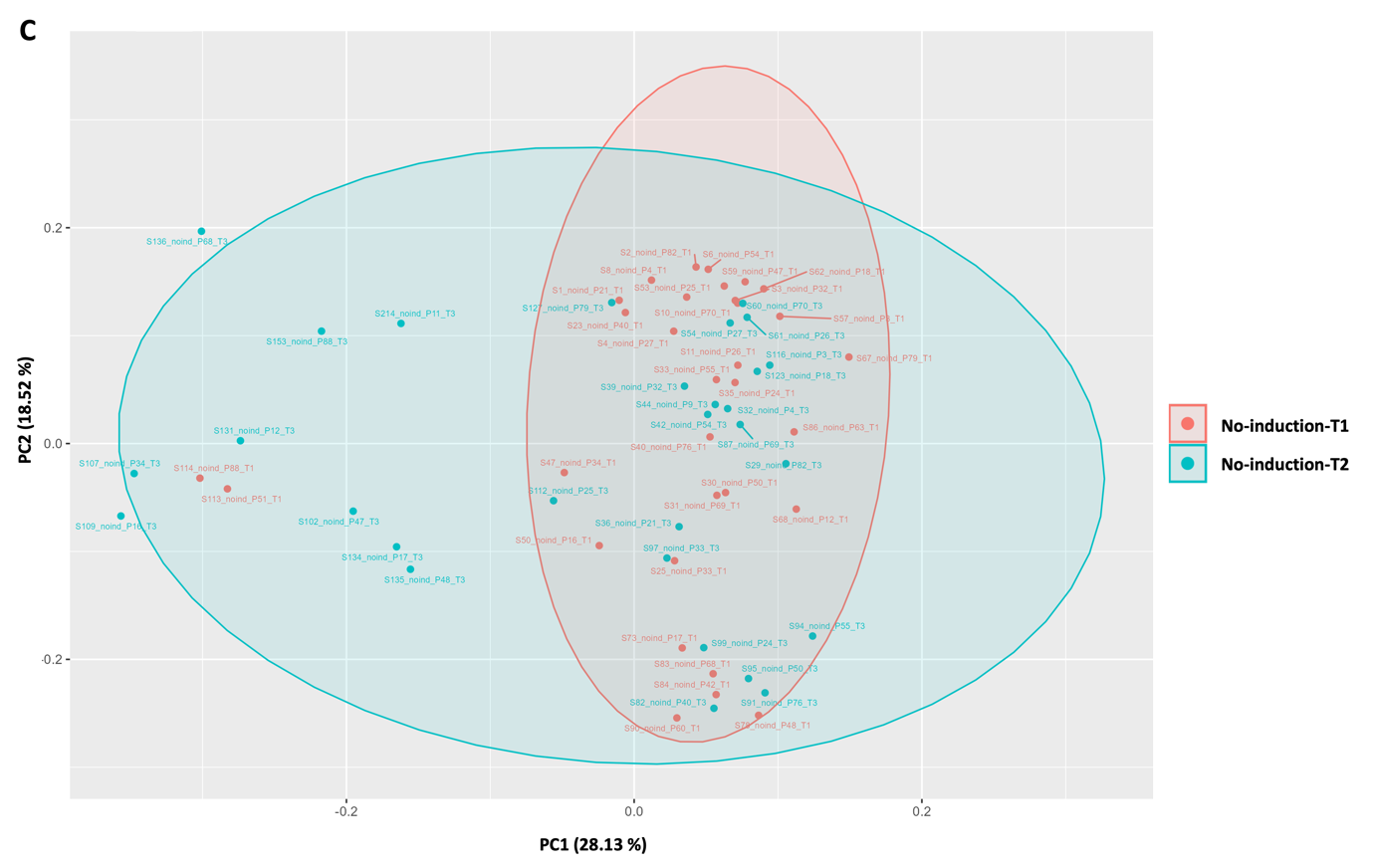

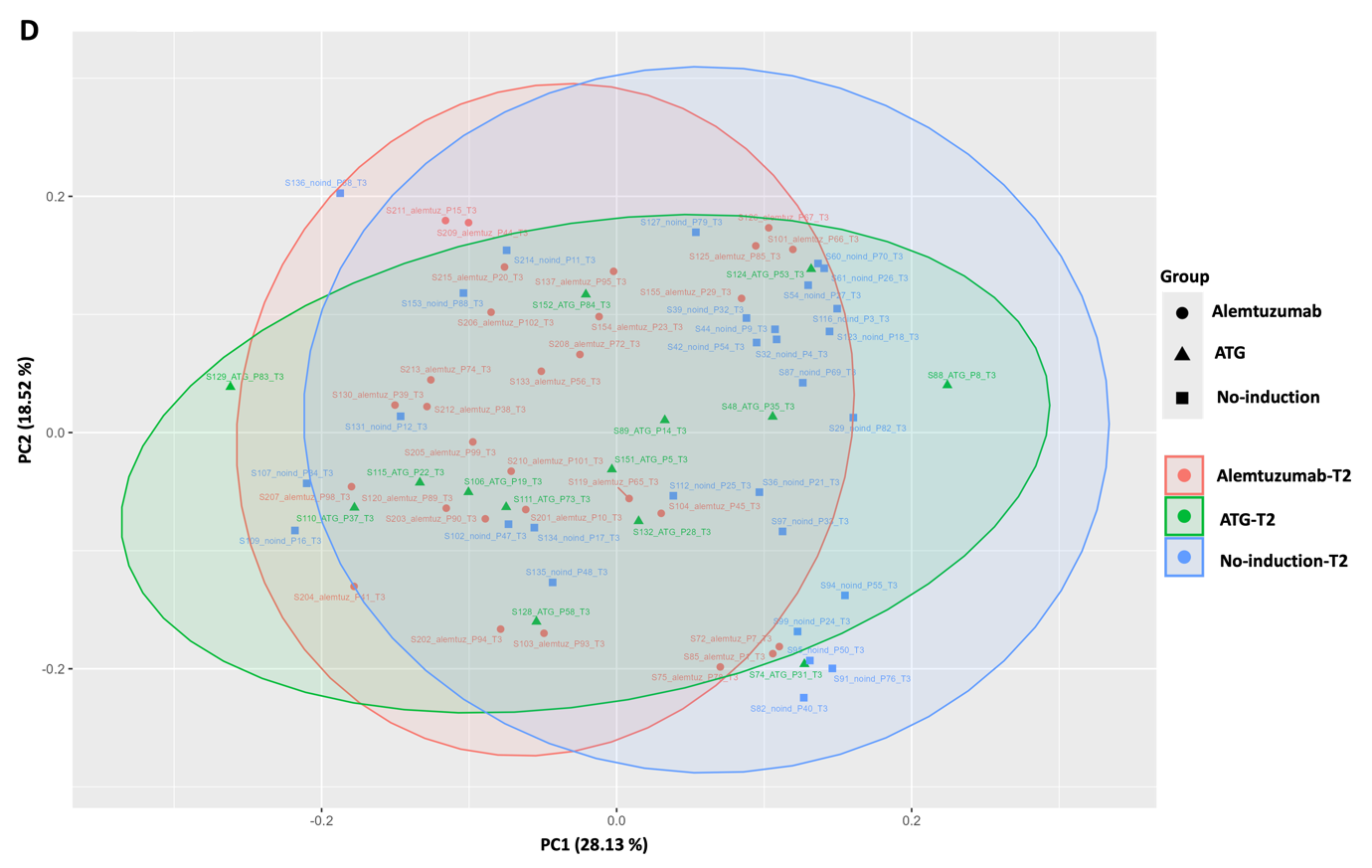

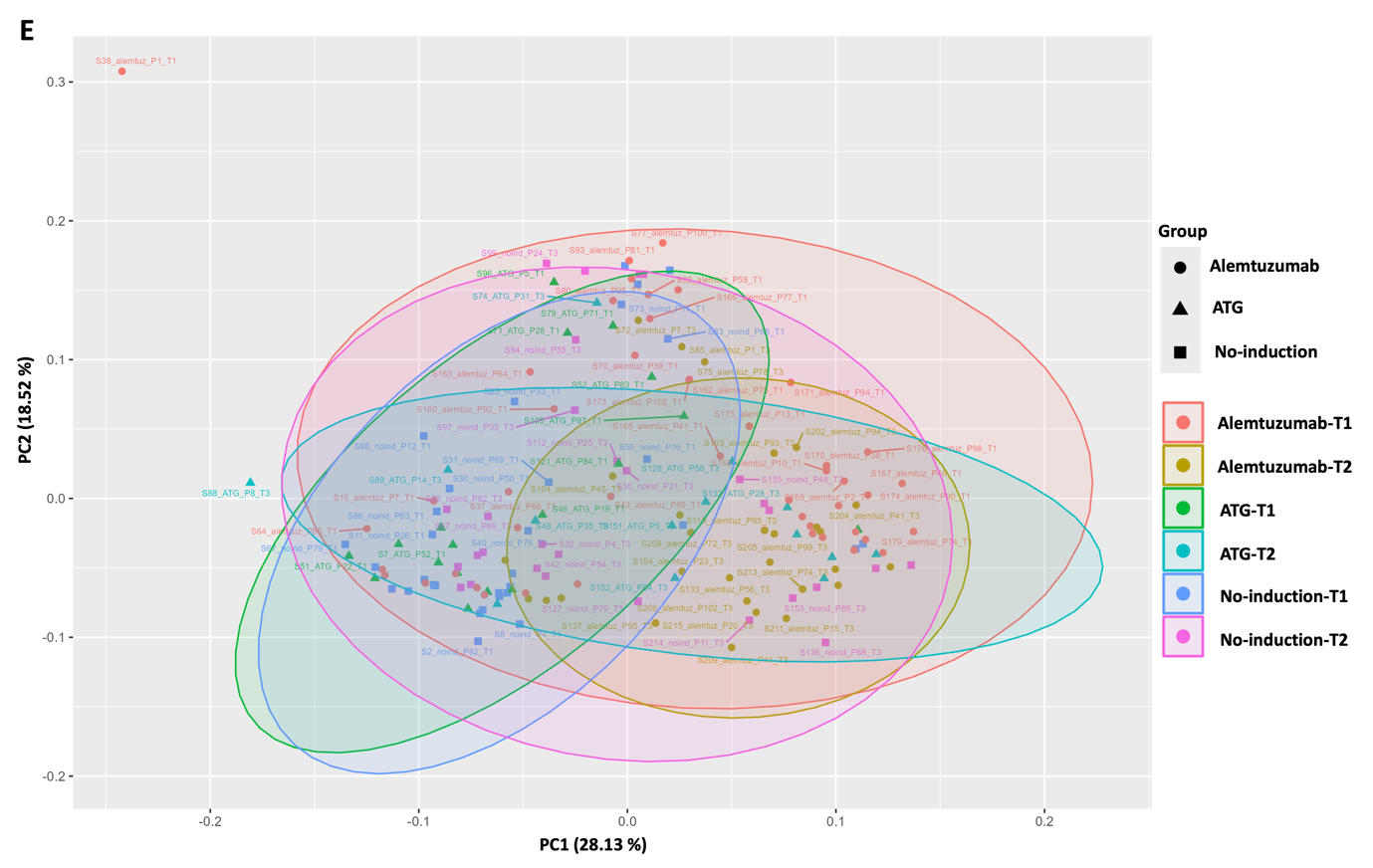
**

**Figure S2. Principal component analysis (PCA) of proteomic profiles. A–C)** Separate PCA plots for each induction group (A: alemtuzumab, B: ATG, C: No‑induction) showing samples at T1 and T2 over time. **D)** Combined PCA plot of all groups at T2. **E)** PCA plots including all samples across all groups and time points.


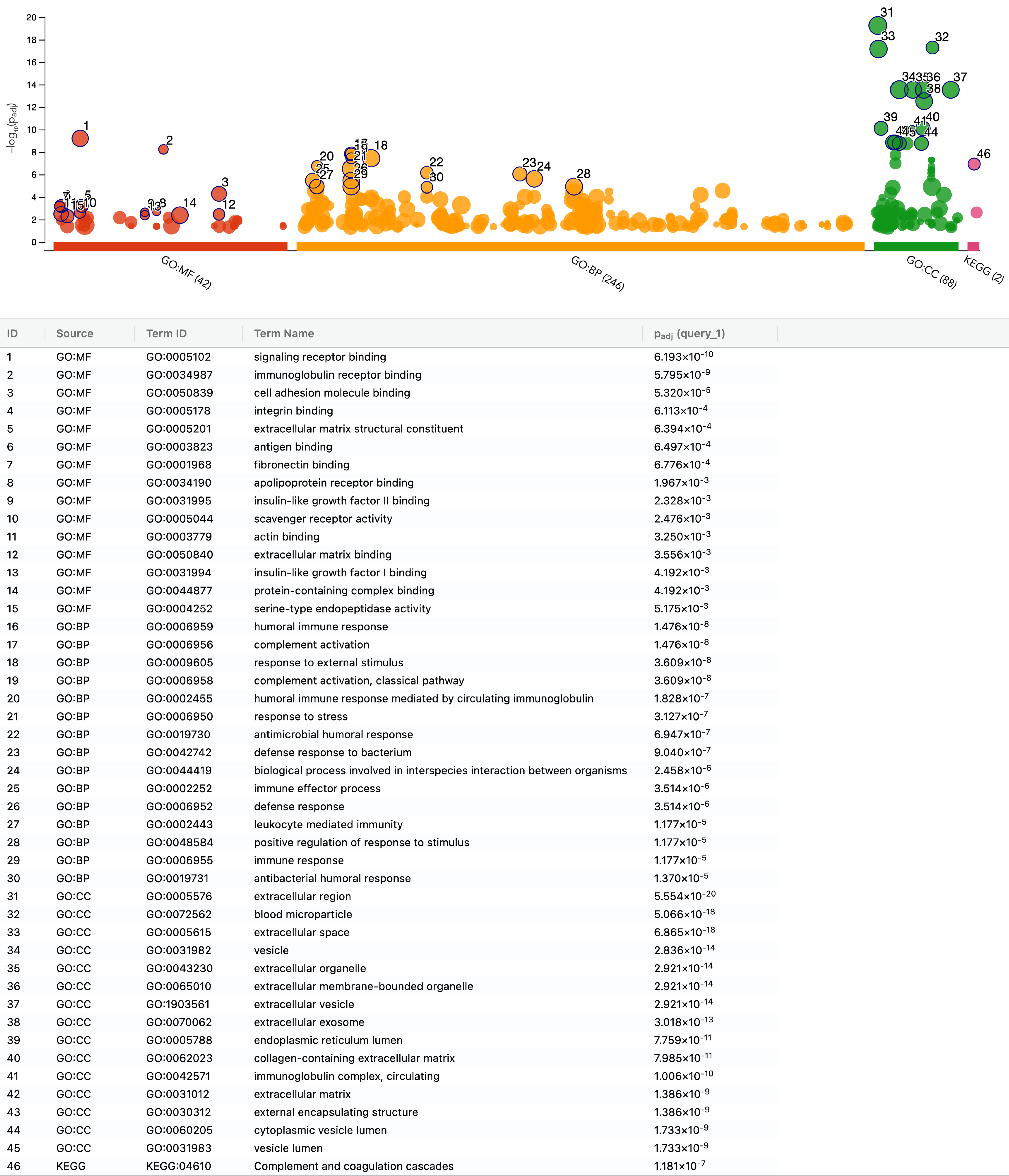
**Figure S3. GO and KEGG pathway enrichment analysis for alemtuzumab T2 vs. alemtuzumab T1**. GO and KEGG pathway enrichment analysis was performed using g:Profiler, separately for significantly upregulated and downregulated proteins identified in the alemtuzumab group between T2 and T1. No significant GO terms or pathways were detected for the upregulated proteins, whereas the downregulated proteins revealed significant enrichment. The dot plot shows enriched GO terms and KEGG pathways identified from the downregulated proteins. The x-axis categorizes terms into molecular function (MF), biological process (BP), cellular component (CC), and KEGG pathways. The y-axis displays the –log10 adjusted p-value, indicating enrichment significance.

The enriched terms predominantly reflect suppressed immune responses, with strong involvement of the humoral immune system, including terms such as signaling receptor binding, immunoglobulin receptor binding, complement activation, and humoral immune response. Cellular localization terms such as extracellular region, vesicle, and exosome suggest reduced abundance of secreted immune effectors. KEGG pathway analysis shows downregulation of complement and coagulation cascades, further supporting suppression of antibody- and complement-mediated immune functions at T2. The top 15 enriched terms from each GO domain (MF, BP, CC) are listed in the accompanying table.


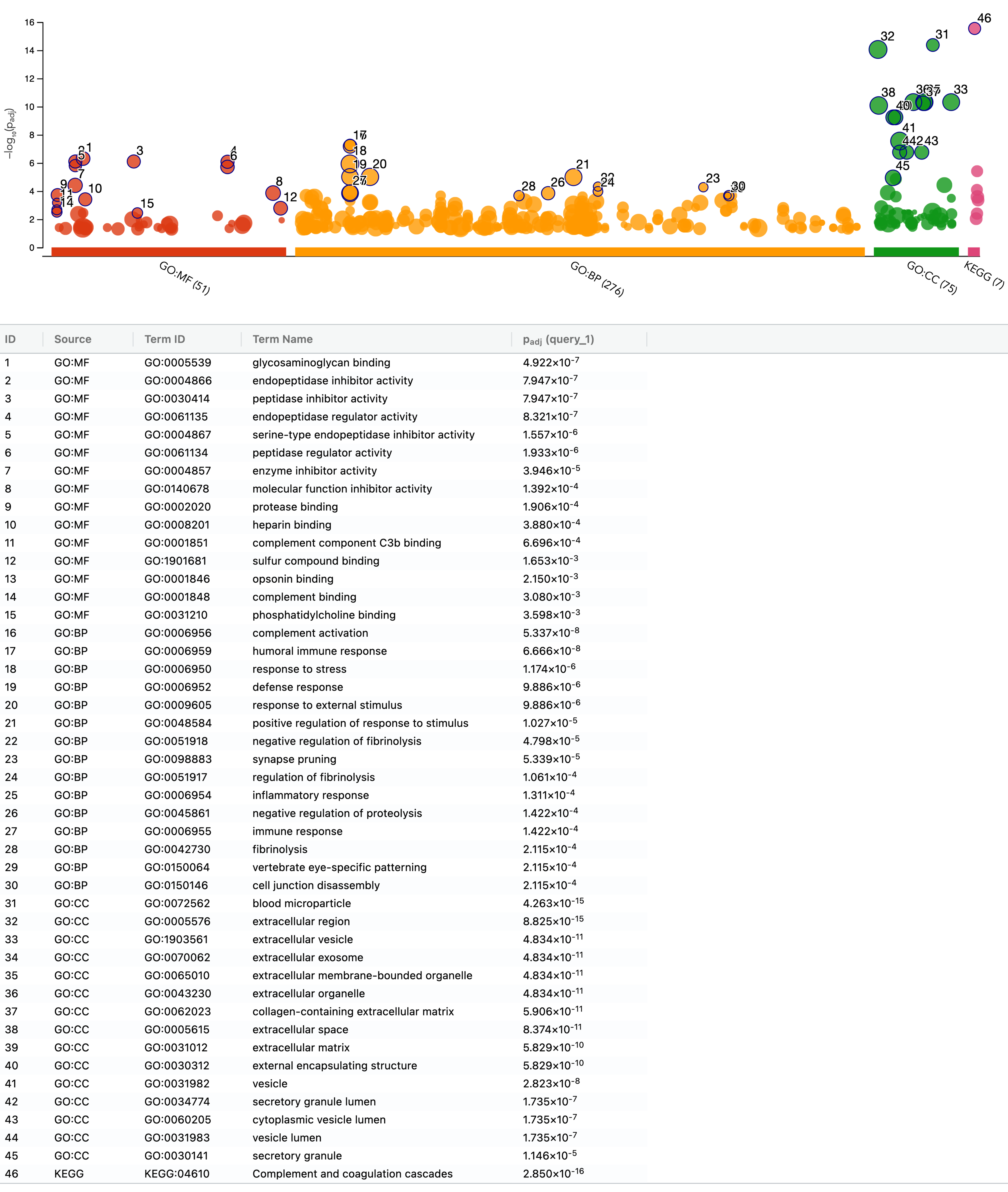
**Figure S4. GO and KEGG pathway enrichment analysis for ATG T2 vs. ATG T1**. GO and KEGG enrichment analysis was conducted using g:Profiler for both upregulated and downregulated proteins in the ATG group between T2 and T1. While no enriched GO terms or pathways were identified for upregulated proteins, the downregulated proteins showed significant functional enrichment, which is presented in this figure. The dot plot visualizes enriched GO terms and KEGG pathways derived from the downregulated protein set. The x-axis categorizes terms into molecular function (MF), biological process (BP), cellular component (CC), and KEGG pathways. The y-axis represents the –log10 adjusted p-value, indicating statistical significance of enrichment. The results reflect suppression of immune and inflammatory processes, including enrichment of terms such as complement activation, response to stress, and inflammatory response. Compared to alemtuzumab, there is less suppression of humoral immune response-related processes, indicating a milder impact on antibody-mediated pathways. KEGG pathway analysis, similarly to alemtuzumab, shows enrichment of complement and coagulation cascades, indicating their downregulation at T2. The top 15 enriched terms from each GO category (MF, BP, CC) are provided in the accompanying table.

**
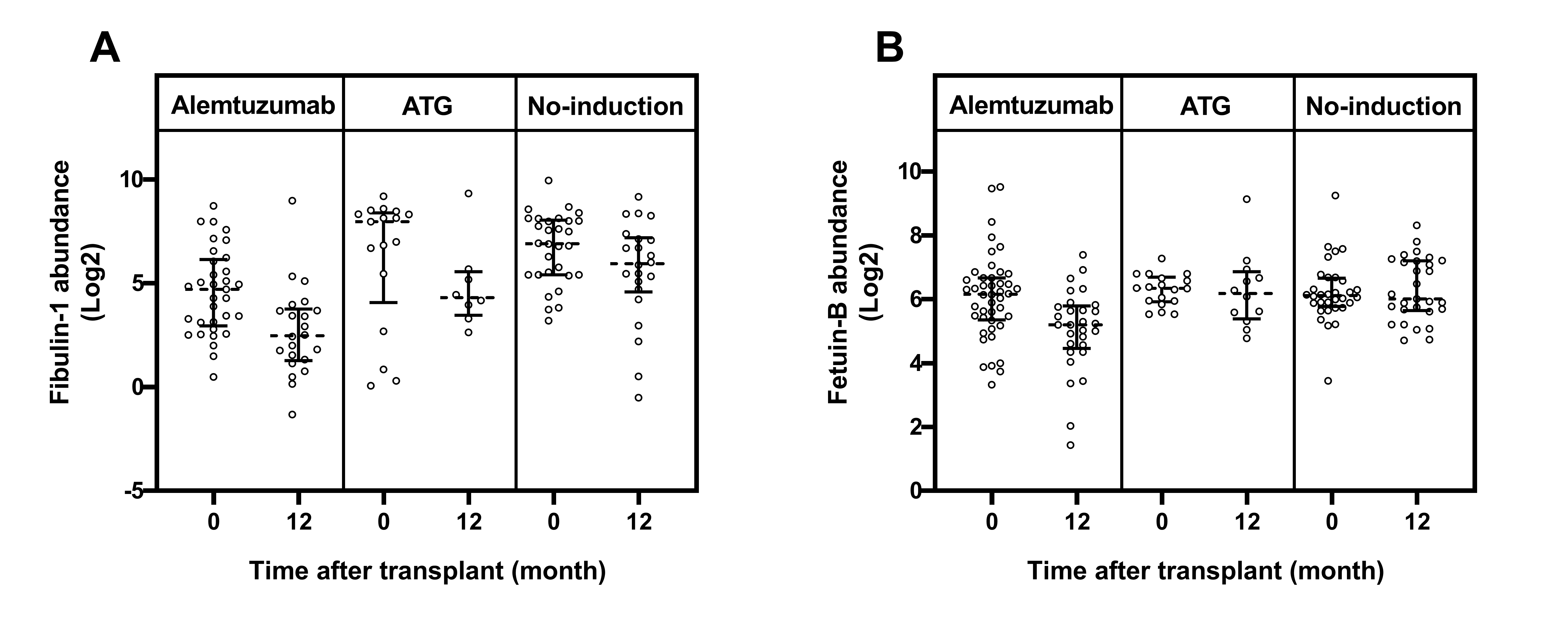
**

**Figure S5. Serum Levels of Fibulin-1 and Fetuin-B in Lung Transplant Recipients.** Serum protein abundances for Fibulin‑1 **(A)** and Fetuin‑B **(B)** are displayed, with each individual sample shown as an open circle. Dashed horizontal lines represent the median for each group and time point; whiskers indicate the interquartile range (25th–75th percentiles). Statistical comparisons were performed using the Mann–Whitney U test**: Fibulin‑1**: alemtuzumab T2 vs T1, p = 0.0015; ATG T2 vs T1, p = 0.1943; no‑induction T2 vs T1, p = 0.1357. **Fetuin‑B:** alemtuzumab T2 vs T1, p = 0.0021; ATG T2 vs T1, p = 0.6539; no‑induction T2 vs T1, p = 0.9457.

**
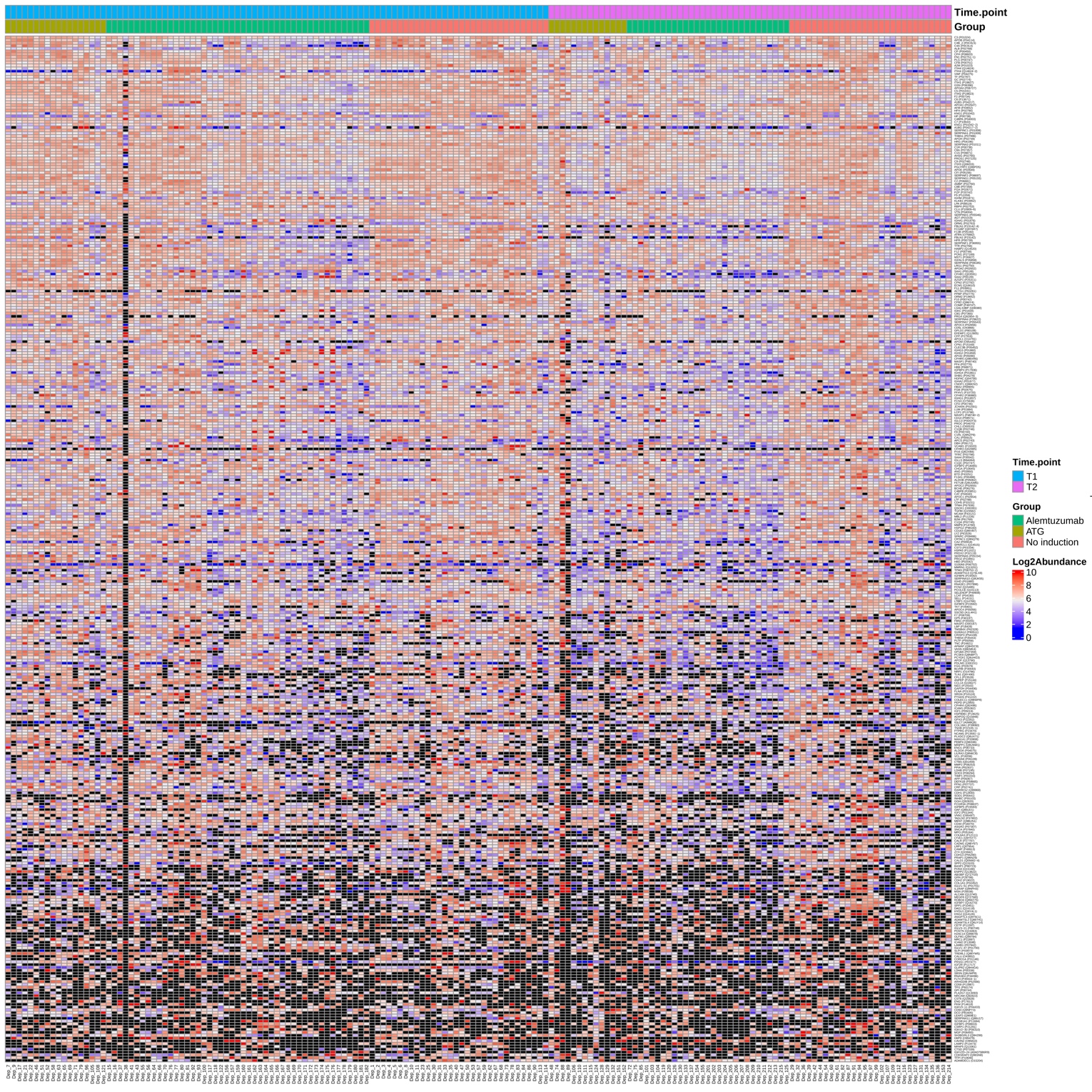
Figure S6. Heatmap of serum protein abundance across all samples, categorized by group and time point.** Columns represent individual patients, and rows correspond to detected proteins (hierarchically clustered). The top bars indicate time point and patient group. Cell colors show log2-transformed protein abundance. Columns are ordered to reflect patient group and time point, allowing visualization of treatment- and time-associated proteomic patterns.

**
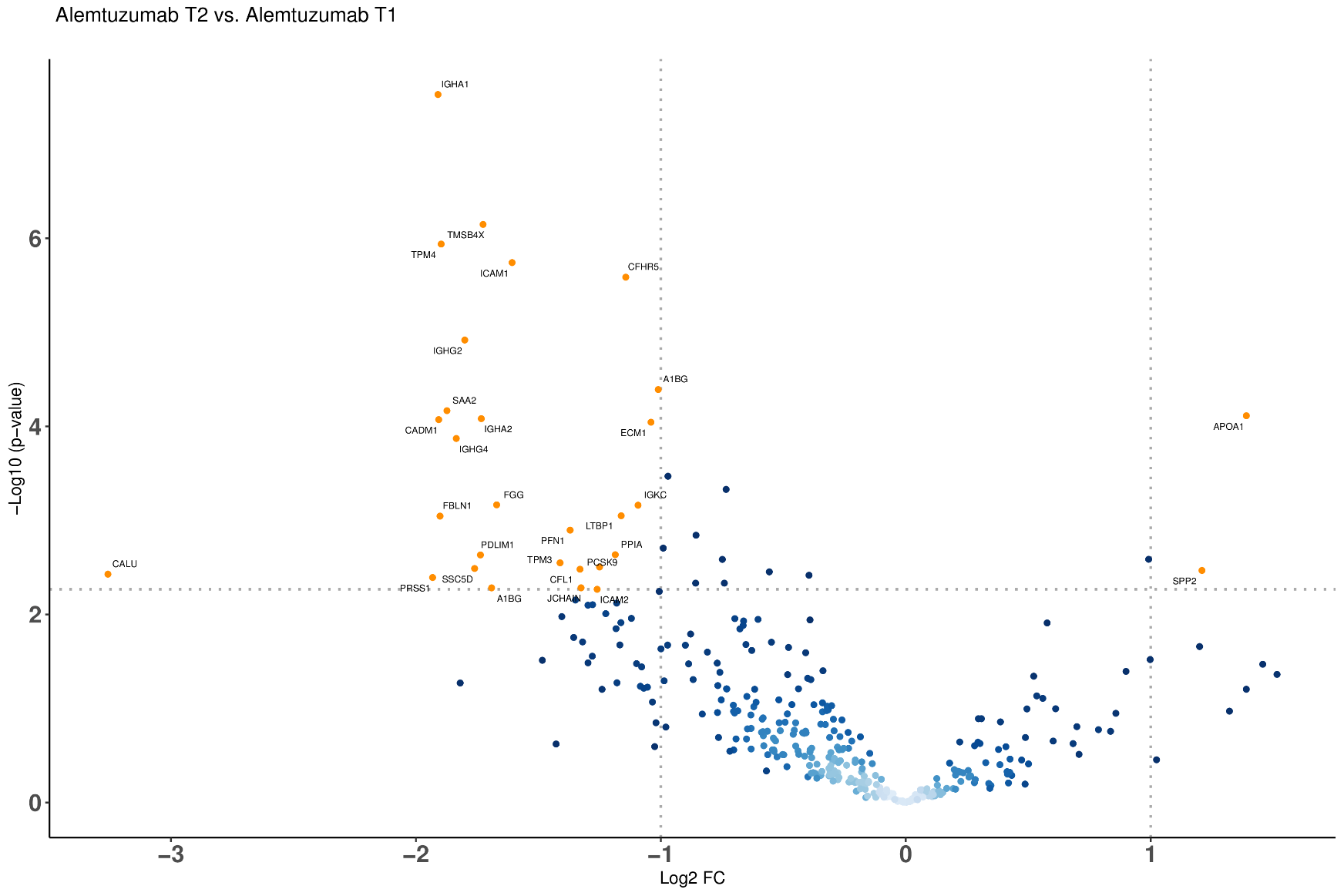
**

**Figure S7. Volcano plot of differentially expressed proteins between alemtuzumab T2 and alemtuzumab T1.** Each dot represents a protein, with log2 fold change (FC) on the x-axis and -log10 raw p-value on the y-axis. Proteins highlighted in orange have Benjamini-Hochberg (BH)-adjusted p-values < 0.05, indicating statistically significant changes in abundance.

**
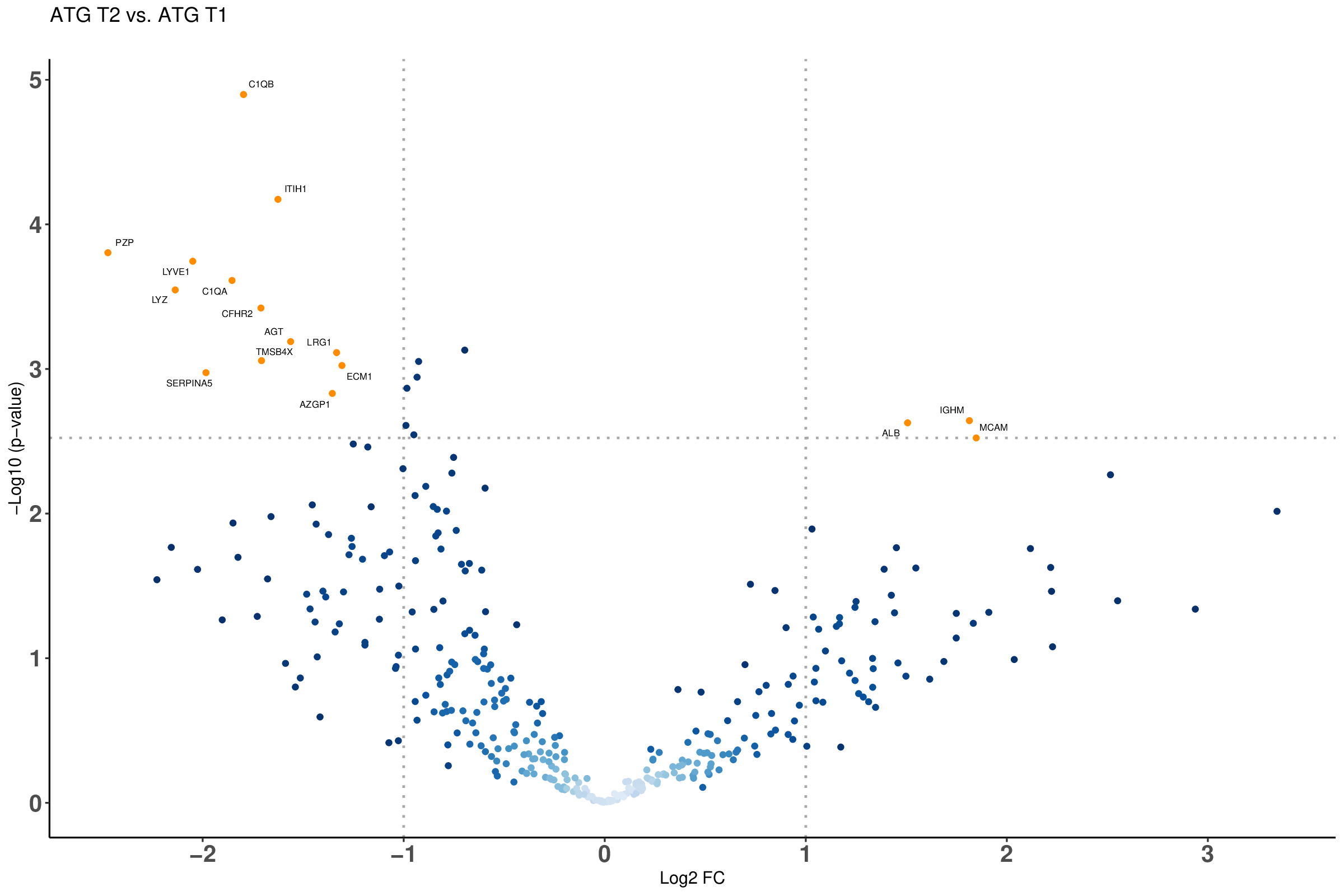
**

**Figure S8. Volcano plot of proteins altered between ATG T2 and ATG T1.** The x‑axis shows log2 fold changes, and the y‑axis displays –log10 raw p‑values. Proteins with Benjamini–Hochberg adjusted p‑values below 0.05 are colored orange and labeled.


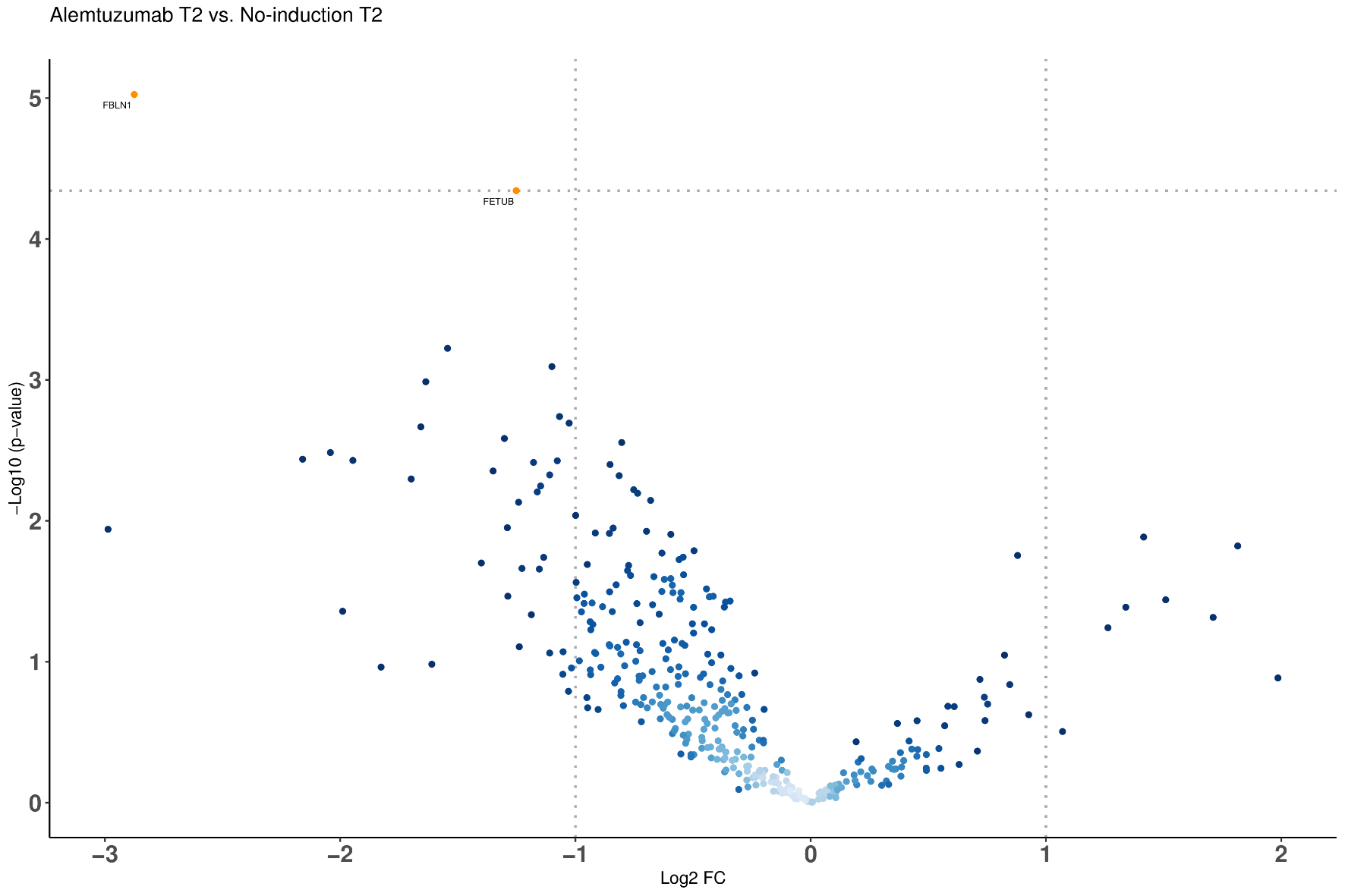


**Figure S9. Volcano plot of differential protein expression between alemtuzumab T2 and No-induction T2.** Log2 fold changes are shown on the x-axis, and –log10 raw p-values on the y-axis. Proteins with Benjamini-Hochberg (BH) adjusted p-values < 0.05 are highlighted in orange.
